# Prenatal valproic acid on the basis of gestational diabetes also induces autistic behavior and disrupts myelination and oligodendroglial maturation slightly in offspring

**DOI:** 10.1038/s41398-025-03450-z

**Published:** 2025-08-07

**Authors:** Maolin Li, Zhifei Qiao, Jizheng Li, Hongli Zhou, Dong Huang, Yan Cai, Xiaolong Li, Zuo Zhang, Jianyun Zhou, Jiyin Zhou

**Affiliations:** 1 Clinical Medical Research Center, The Second Affiliated Hospital, Army Medical University (Third Military Medical University), Chongqing, China; 2 Department of Orthopedics, First Clinical Medical College of Yunnan University of Traditional Chinese Medicine, Kunming, China

**Keywords:** Molecular neuroscience, Human behaviour

## Abstract

**Introduction:**

Gestational diabetes mellitus (GDM) and prenatal exposure to valproic acid (VPA) are both constitute risk factors for autism in progeny. Notably, dysmyelination in the corpus callosum serves as a prominent element connecting GDM and autism in the white matter lesions.

**Objective:**

The cumulative effects of GDM and prenatal VPA on both autistic behavior and dysmyelination in progeny have been investigated in this study.

**Methods:**

In vivo, female mice exhibiting leptin receptor deficiencies and maintained on a high-fat diet were utilized to create GDM models, to which prenatal VPA was administered. In vitro, oligodendrocyte precursor cells (OPCs) were treated with VPA in the high-fat and high-glucose culture.

**Results:**

The offspring subjected to both GDM and prenatal VPA demonstrated comparable declines in social interaction, myelination, and OPC maturation, akin to those exclusively exposed to VPA. Remarkably, the application of clemastine facilitated remyelination, ameliorated autistic behaviors, and promoted the progression of OPCs. Furthermore, the compromised myelination and OPC maturation instigated by the combination of GDM and prenatal VPA were found to be less severe compared to those precipitated by VPA alone. This differential impact can be attributed to the opposing influences of GDM and VPA on gamma-aminobutyric acid receptor activation in OPCs, extracellular regulated protein kinases (ERK) phosphorylation in OPCs, and the modulation of histone deacetylase 3 and dual specificity phosphatase 5 expression.

**Conclusions:**

we delineate the antagonistic effects of GDM and prenatal VPA on ERK phosphorylation in fetal OPCs, consequently altering their proliferation and differentiation, thereby culminating in milder dysmyelination and autistic behaviors.

## Introduction

Epilepsy during pregnancy, a significant contributor to fetal growth restriction, remains difficult to manage with drugs that are both effective and have low toxicity [1]. Valproic acid (VPA), widely utilized to treat hereditary epilepsy and non-epileptic disorders such as migraines, has proven to be efficacious [2, 3]. Although the FDA regards VPA as potentially harmful to the fetus, its usage in low doses is permissible owing to its benefits in managing epilepsy during pregnancy [4]. Animal studies frequently employ prenatal VPA administration to induce autism spectrum disorders (ASD) in offspring [5], a process that influences neural proliferation and differentiation, axonal growth and material transport, and histone acetylation in the central nervous system (CNS) [6]. Moreover, autistic fetuses and infants often demonstrate CNS dysfunctions associated with numerous metabolic abnormalities occurring during pregnancy [7, 8]. Clinical data indicates a pronounced impact of gestational diabetes mellitus (GDM) on the increased risk of autism in offspring [9, 10]. Offspring from heterozygous leptin receptor-deficient (db/−) female mice experiencing hyperglycemia during pregnancy [11], and those from mice on a high-fat diet during gestation [12], present phenotypes similar to autism. These autistic phenotypes are purported to be induced by persistent production of reactive oxygen species, oxidative stress-mediated hindrance of histone methylation, and heightened expression of superoxide dismutase 2 in the amygdala during prenatal hyperglycemia [13]. Furthermore, significant mediation also occurs through oxytocin receptor inhibition in the hypothalamus and hippocampus [14]. Concurrently, inflammatory reactions and alterations in histone methylation in the amygdala, hypothalamus, and hippocampus contribute to autistic behaviors seen in cases of GDM-associated vitamin D deficiency [15].

Myelin, a critical structural component, facilitates swift neural communication and the rapid conduction of action potentials in the CNS [16–18]. Postnatal myelination transpires as myelin sheaths encase nerve fibers [19, 20], involving the proliferation, migration, and differentiation of oligodendrocyte precursor cells (OPC), alongside the envelopment and folding processes carried out by mature oligodendrocytes (OL) [21]. Frequently, animal models utilize the corpus callosum (CC) for studying myelination disruption, given its status as the region with the highest concentration of myelinated nerve fibers in the CNS [22]. Notably, disrupted corpus callosum integrity and aberrant white matter microstructures are observed in models of autism [23, 24] or in offspring impacted by GDM [25]. Animal models of ASD exhibit various compromised dynamic processes linked to myelination deficits in the CC [26–28], encompassing hindered OPC maturation [29], diminished mature OLs [30], and escalated OPC proliferation [31]. GDM disrupts callosal myelination, activated by type 2 voltage-gated chloride channels and the inhibition of serine/threonine-protein kinase, thereby precipitating OL oxidative stress and apoptosis [32].

GDM and prenatal VPA exhibit analogous effects in fostering autistic phenotypes in offspring, notably affecting the microstructure of white matter and inhibiting myelination in the CC. The concurrent implications of GDM and prenatal VPA exposure remain underexplored. Previous animal research primarily centered on the impacts on neurons and neural stem cells in the fetal brain, with studies pertaining to myelination and oligodendrocyte lineage development still in nascent stages. In this investigation, we amalgamated prenatal VPA and GDM models to delineate the distinctions in offspring growth and social behavior attributable to both combined and isolated factors. Moreover, we have fostered new perspectives on dysmyelination in the CC and variations in the proliferation and differentiation of the OPC to elucidate the influence of GDM and prenatal VPA on fetal brain development. This research extends novel insight for managing patients affected by both GDM and epilepsy who are undergoing VPA treatment, addressing potential deficits in CNS, albeit in rare instances. Furthermore, this research proposes a novel animal experimental model to scrutinize the associations between VPA and ASD.

## Methods and materials

### Key resource table

The details are in Table 1.

**Table 1:** Key resource table.

No.	Reagent type (species) or resource	Designation	Source or reference	Identifiers	Additional information	Nationality
1	Antibody	Anti-CNPase antibody [1H10]	Abcam	ab277621	Immunohistochemistry, (1:400)	USA
2	Antibody	Myelin Basic Protein (D8X4Q) XP**®** Rabbit mAb	Cell Signaling Technology	78896 s	Immunohistochemistry, (1:800)	USA
3	Antibody	Olig2 (E6G6Q) XP**®** Rabbit mAb	Cell Signaling Technology	65915S	Immunofluorescence, (1:200)	USA
4	Antibody	CD140a (PDGFRA) Monoclonal Antibody (APA5)	Thermo Fisher Scientific	12140181	Immunofluorescence, (1:100)	USA
5	Antibody	Anti-APC (Ab-7) Mouse mAb (CC-1)	Millipore	OP80	Immunofluorescence, (1:50)	USA
6	Antibody	Anti-PDGFR alpha antibody [EPR22059-270]	Abcam	ab203491	Immunofluorescence, (1:100)	USA
7	Antibody	Anti-PCNA antibody [PC10]	Abcam	ab29	Immunofluorescence, (1:100)	USA
8	Antibody	Phospho-p44/42 MAPK (Erk1/2) (Thr202/Tyr204) (D13.14.4E) XP**®** Rabbit mAb	Cell Signaling Technology	4370 T	Immunofluorescence, (1:100)	
Immunoblotting, 1:1000	USA					
9	Antibody	Anti-NG2 Chondroitin Sulfate Proteoglycan, clone 132.38	Millipore	mab5384-I	Immunofluorescence, (1:200)	USA
10	Antibody	HDAC3 (D2O1K) Rabbit mAb	Cell Signaling Technology	85057S	Immunoblotting, (1:1000)	USA
11	Antibody	beta-Actin Monoclonal Antibody (15G5A11/E2)	Introvergen	MA1-140	Immunoblotting, (1:3000)	USA
12	Antibody	beta Tubulin Loading Control Monoclonal Antibody (BT7R)	Introvergen	MA5-16308	Immunoblotting, (1:3000)	USA
13	Antibody	Lamin B1 antibody	GeneTex	GTX103292	Immunoblotting, (1:1000)	USA
14	Antibody	p44/42 MAPK (Erk1/2) (137F5) Rabbit mAb	Cell Signaling Technology	4695 T	Immunofluorescence, (1:100) Immunoblotting, (1:1000)	USA
15	Antibody	HDAC1 (D5C6U) XP**®** Rabbit mAb	Cell Signaling Technology	34589 T	Immunoblotting, (1:1000)	USA
16	Antibody	HDAC2 Rabbit Monoclonal Antibody	Beyotime	AF1555	Immunoblotting, (1:1000)	China
17	Antibody	HDAC8 Rabbit Monoclonal Antibody	Beyotime	AF2737	Immunoblotting, (1:1000)	China
18	Antibody	MEK1/2 (D1A5) Rabbit mAb	Cell Signaling Technology	8727 T	Immunoblotting, (1:1000)	USA
19	Antibody	mTOR (7C10) Rabbit mAb	Cell Signaling Technology	2983 T	Immunoblotting, (1:1000)	USA
20	Antibody	Phospho-mTOR (Ser2448) (49F9) Rabbit mAb	Cell Signaling Technology	2976S	Immunoblotting, (1:1000)	USA
21	Antibody	Akt (pan) (11E7) Rabbit mAb	Cell Signaling Technology	4685S	Immunoblotting, (1:1000)	USA
22	Antibody	Phospho-Akt (Ser473) (D9E) XP**®** Rabbit mAb	Cell Signaling Technology	4060 T	Immunoblotting, (1:1000)	USA
23	Antibody	Alexa Fluor 647-labeled Goat Anti-Rabbit IgG	Beyotime	A0468	Immunofluorescence, (1:500)	China
24	Antibody	Alexa Fluor^TM^ 488 Goat anti-Rabbit IgG	Introvergen	A11008	Immunofluorescence, (1:500)	USA
25	Antibody	Alexa Fluor^TM^ 488 Goat anti-Rat IgG	Introvergen	A11006	Immunofluorescence, (1:500)	USA
26	Antibody	Alexa Fluor Cy3-labeled Goat Anti-Mouse IgG	Beyotime	A0521	Immunofluorescence, (1:500)	China
27	Antibody	Alexa Fluor 488^®^ Goat Anti-Rabbit IgG H&L	Abcam	ab150077	Immunofluorescence, (1:1000)	USA
28	Antibody	HRP-labeled Goat Anti-Rabbit IgG(H+L)	Beyotime	A0208		China
29	Antibody	HRP-labeled Goat Anti-Mouse IgG(H+L)	Beyotime	A0216		China
30	Kit	Cell Counting Kit-8	Dojindo	CT04-100		Japan
31	Kit	Universal mouse/rabbit polymer method detection system	ZSGB Biotech	PV-6000		China
32	Kit	DAB chromogenic kit	ZSGB Biotech	ZLI-9019		China
33	Kit	Nuclear protein extraction kit	Solarbio	R0050		China
34	Kit	Bicinchoninic acid protein assay kit	Beyotime	P0012		China
35	Kit	BeyoECL star kit	Beyotime	P0018AS		China
36	Kit	Luxol fast blue staining kit	Solarbio	G3245		China
37	Cell line	OLN-93	Yaji Biotechnology	YS1232C		China
38	Serum	Fetal Bovine Serum	Lonsera	S711-001S	10%	Uruguay
39	Formula feeds	High-fat diet (45 kcal/day)	Medicience Biotechnology	MD12032		China
40	Reagent	DMEM	Procell Life Science & Technology	PM150210		China
41	Reagent	Penicillin-streptomycin Premix	Beyotime	C0222	1%	China
42	Reagent	Sodium Valproate	Solarbio	IV0010	Mice, 600 mg/Kg Cells, 5 mM	China
43	Reagent	Clemastine	Institute of Food and Drug Control	ALH8	Mice, 10 mg/Kg	China
44	Reagent	DMSO	Solarbio	D8371		China
45	Reagent	(+)-Glucose	BBI Lifescience	A600219	Cells, 50 mM	China
46	Reagent	Gamma-aminobutyric Acid	Sigma	A2129	Cells, 100 mM	USA
47	Reagent	(+)-Bicuculline	Selleck	S7071	Cells, 100 μM	China
48	Reagent	CGP52432	Selleck	S0303	Cells, 25 μM	China
49	Reagent	U0126	Selleck	S1102	Cells, 10 μM	China
50	Reagent	Palmitic acid	Selleck	S3794	Cells, 200 μM	China
51	Reagent	Antifade mounting medium with DAPI	Beyotime	P0131		China
52	Reagent	fat-free BSA	Biofroxx Biotech		Cells, 200 μM	China
53	Reagent	Sodium Pentobarbital	Sigma	P3636	1%	USA
54	Reagent	Paraformaldehyde Solution	Servicebio	G1101	4%	China
55	Reagent	Glutaraldehyde Solution	Servicebio	G1124	2.50%	China
56	Reagent	RIPA lysis buffer	Beyotime	P0013B		China
57	Reagent	Phosphatase inhibitor cocktail A	Beyotime	P1082		China
58	Reagent	Protease inhibitor cocktail	Beyotime	P1008		China
59	Reagent	EDTA	Beyotime	P1005		China
60	Reagent	SDS-PAGE sample loading buffer	Beyotime	P0015		China
61	Reagent	Quick antigen retrieval solution for frozen sections	Beyotime	P0090		China
62	Reagent	Goat serum solution	Solarbio	SL038	10%	China
63	Reagent	Western rapid transfer buffer	Beyotime	P0572		China
64	Reagent	BeyoGel^TM^ plus SDS-PAGE hepes electrophoresis buffer	Beyotime	P0552		China
65	Reagent	Wb antigen blocking and antibody sensitization diluent	Willget Biotech	F01		China
66	Materials	BeyoGel^TM^ plus precast PAGE gel	Beyotime	P0452	Tris-Gly, 8%, 15 wells	China
67	Materials	PVDF membranes	Millipore	ISEQ00010		USA
68	Markers	PageRuler^TM^ Prestained Protein Ladder	Thermo Fisher Scientific	26616		USA
69	Markers	PageRuler^TM^ Prestained Protein Ladder	Thermo Fisher Scientific	26619		USA

### Animals and OPC culture

A total of one hundred female mice, subdivided into two distinct strains - fifty SPF C57BL/6J mice and fifty SPF C57B6.Cg-Dock7 +/− Lepr /J mice, both aged between 8–10 weeks and with respective body weights of 20 ± 2 g and 21 ± 2 g, were procured for this study. The former group was sourced from the Animal Experiment Center of Xinqiao Hospital, Army Medical University, while the latter was self-bred, originating from breeder specimens acquired from Jackson Laboratories, USA. All mice were housed under controlled conditions featuring a temperature of 22 ± 2 °C, a relative humidity of 55 ± 5%, and a 12 h light-dark cycle, with ad libitum access to water.

In vitro assays employed immature oligodendrocytes OLN-93, a readily passagable OPC cell line, cultivated according to the protocol described by Strelau et al. [33]. The cells were maintained in a 5% CO_2_ incubator at 37 °C, grown in DMEM fortified with 10% fetal bovine serum and 1% penicillin-streptomycin. These cells were subcultured at a (1:2) ratio every three days, with cells from the 6th to 12th generations being selected for experimental procedures.

### Materials and model induction

The sodium valproate, a soluble short-chain fatty acid, was dissolved in saline to create a 100 mg/ml solution and was stored at −20 °C, protected from light, in preparation for animal experiments. Clemastine, known for its promyelinating properties, was prepared at a concentration of 35 mg/ml using DMSO as the solvent. For the cellular studies, VPA was initially dissolved in DMSO to obtain a 2.5 M solution. This stock solution was later diluted with DMEM, ensuring the final DMSO concentration did not surpass 1% of the total mixture. Palmitic acid solution (20 mM) was prepared according Xin et al. [34]. and utilized for OLN-93. Glucose and gamma-aminobutyric acid (GABA) were dissolved directly in DMEM to obtain a 5 M glucose stock solution and a 1 M GABA stock solution. (+)-Bicuculline (GABA_A_ receptor antagonist), CGP52432 (GABA_B_ receptor antagonist) and U0126 (ERK inhibitor) were all solubilized in DMSO to 10 mM and stored them at −20 °C away from light.

In the experiment, fifty female C57BL6 mice in estrus were identified, numbered, and randomly assigned into either the control group (*n* = *25*) or VPA group (*n* = *25*). An additional fifty female C57B6.Cg-Dock7 +/− Lepr /J mice were likewise identified, numbered, and bifurcated randomly into GDM group (*n* = *25*) and GDM+VPA group (*n* = *25*). Mating was facilitated by housing male mice of corresponding genotypes with the females at a ratio of (1:2) for a period of 72 h. Fertilization success was confirmed through the identification of a vaginal plug, marking the initiation of the embryonic timeline at day 0.5 (E+0.5d). Subsequently, on day 12.5 of embryonic development (E+12.5d), mice in the VPA and GDM+VPA groups were administered a subcutaneous injection of 0.25 ml of a saline-diluted VPA solution at a dose of 600 mg/kg [35]. Meanwhile, the control group and GDM group were given a 0.25 ml subcutaneous injection of saline. To induce gestational diabetes, the GDM and GDM+VPA groups were subjected to a continuous high-fat diet regimen supplying 45 kcal/day, starting seven days prior to the embryonic timeline (E-7 d) and maintained throughout the pregnancy. Following birth, the offspring were categorized into four groups mirroring their respective maternal groupings: control, VPA, GDM, and GDM+VPA.

To investigate the potential dysmyelination in offspring induced by the promyelinating agent clemastine, pups from different litters were segregated at postnatal day (PND) 28 into four groups: the control group (*n* = *8*), the clemastine group (*n* = *8*), the VPA group (*n* = *8*), and the VPA+clemastine group (*n* = *8*), derived from both control and VPA groups. Saline served as the placebo, while the clemastine and VPA+clemastine groups received an intraperitoneal injection of 10 mg/kg saline-diluted clemastine continuously for 21 days, commencing from the peak of myelination in the corpus callosum [20, 36].

In the in vitro experiments, GABA, VPA, (+)-Bicuculline, and CGP52432 were all diluted in cell growth medium prior to used. The effects of various concentrations of these drugs on OLN-93 cells were assessed using the Cell Counting Kit-8 (CCK-8) cell activity assay to evaluate cell activity. Following a 12 h period to allow for complete adhesion, the OLN-93 cells were exposed to 100 mM GABA for 24 h to activate cellular GABA receptors. Subsequently, 100 μM (+)-Bicuculline and 25 μM CGP52432 were administered as positive controls to demonstrate GABA receptor antagonism, with the results compared to those obtained following a 24 h treatment with 0.5 mM VPA.

In order to mimic the conditions of GDM-induced injury, an in vitro high-fat, high-glucose (HFHG) culture environment was established using 200 μM palmitic acid and 50 mM glucose, according to the protocol outlined by Liu et al. [37]. Under these conditions, OLN-93 cells were subjected to treatments with GABA, VPA, and the established positive controls once more. Furthermore, to analyze the repercussions of inhibiting ERK signaling in oligodendrocyte precursor (OPC) cells, the fully adhesive OLN-93 cells were initially treated with 10 μM U0126 for 30 min. The earlier established GABA and VPA treatment protocols were then replicated under both normal and HFHG conditions to observe any consequent alterations.

### Maternal blood glucose and development of offspring

On pre-embryonic day 7 and embryonic day 0.5, 7.5, 15.5, and 21.5 (E-7 d, E+0.5d, E+7.5d, E+15.5d, E+21.5d), the pregnant mice were weighed. The second drop of blood was collected from the tip of the tail to measure fasting blood glucose levels using a glucometer (Sinocare, GA-3, China), following 8 h of food and water deprivation. On E+12.5d, the oral glucose tolerance test (OGTT) was conducted in accordance with standard protocols after an 8 h period of food and water deprivation. The pups’ blood glucose levels and body weight were monitored at PND 28, 42, and 56. The development of the pups between PND 8 and PND 12 was assessed using eye-opening scores and orientation tests.

### Social behavior

**Fig. 1:**
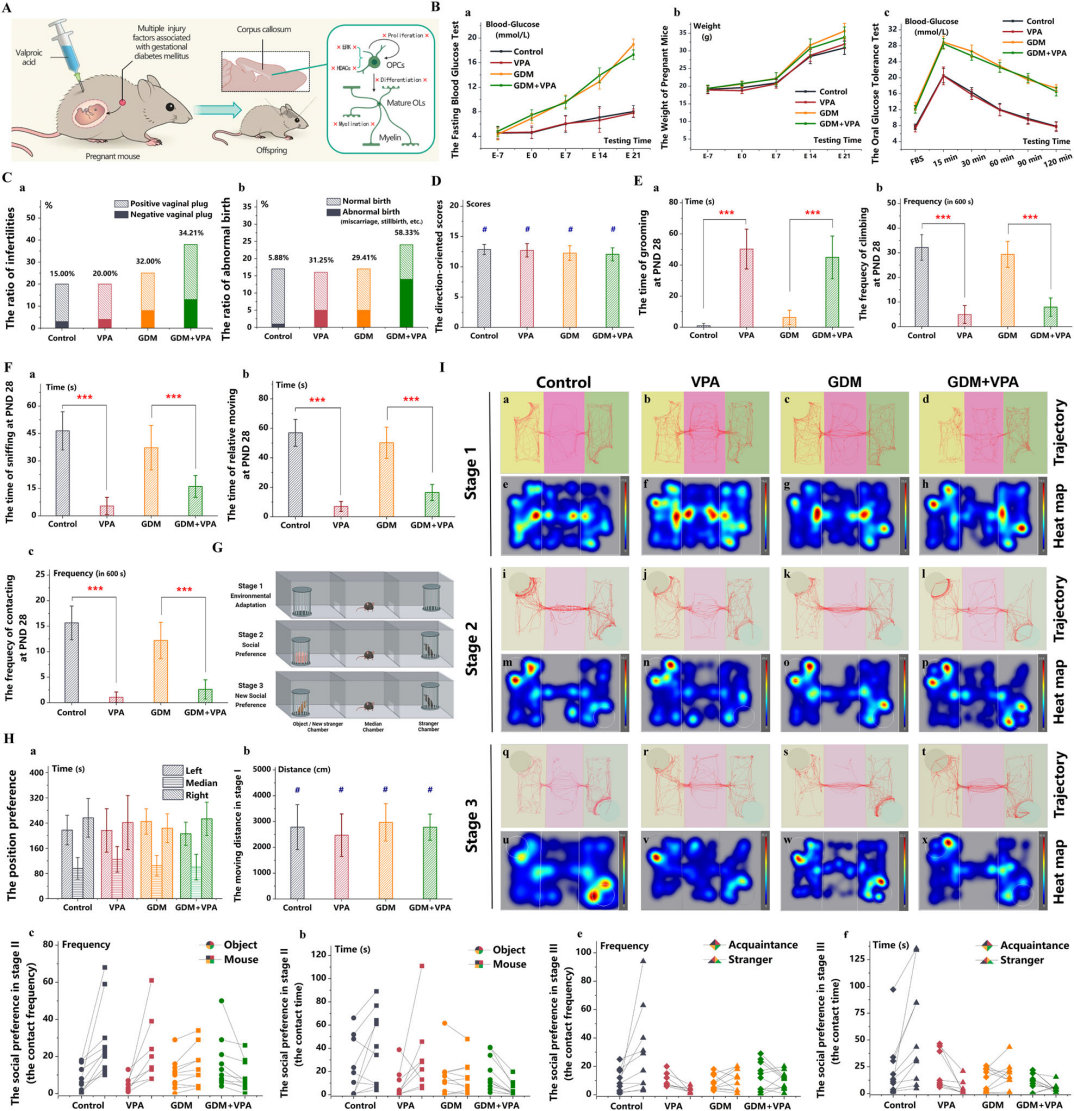
Offspring exposed to GDM and prenatal VPA also exhibit social impairments. **A** The GDM+VPA animal model was established with a 45 kcal% high-fat diet and a subcutaneous VPA injection on E+12.5d in female mice with heterozygous leptin receptor deficiencies. **B** The fasting blood glucose **a**, the oral glucose tolerance test **b** and the body weight **c** changed in control, VPA, GDM, and GDM+VPA groups (*n* = *10*). **C** The rates of reproductive failure **a** and adverse pregnancy outcomes **b**, such as abortion and stillbirth, in control, VPA, GDM, and GDM+VPA groups. **D** The direction-oriented scores indicating postnatal brain development in four progeny groups: control, VPA, GDM, and GDM+VPA (*n* = *10*). **E** The repetitive and stereotypical patterns of behavior reflected by the grooming time **a** and the frequency of exploration **b** during the open field test in four progeny groups (*n* = *10*). **F** The social interactions in the PND 28 pups encompassed behaviors such as sniffing **a**, following **b**, and propelling **c** (*n* = *10*). **G** the diagram of the three-chamber sociability test (*n* = *10*). **H** PND 56 pups’ location preferences **a** and athletic ability **b** were analyzed in the initial phase of the three-chamber test. In the subsequent stages, the preference for an object over a new mouse was measured by contact time **c** and frequency **d** among PND 56 pups. The contact time **e** and frequency **f**, which reflected the preference for a strange mouse over a familiar mouse, was measured in the final stage of the test. **I** PND 56 pups’ trajectory (**a**–**d,** **i**–**l**, and **q**–**t**) and heat maps (**e**–**h,** **m**–**p**, and **u**–**x**) in three stages in the three-chamber test. ***P < 0.001, control group compared with VPA group, or GDM group compared with GDM + VPA group. ^#^*P* > 0.05, control group compared with VPA group, or GDM group compared with GDM + VPA group.

As per the methodology outlined by Cohen et al. [38], repetitive/stereotypical behaviors, juvenile reciprocal social interactions, and three-chamber sociability were evaluated using the Noldus EthoVision-XT animal motion tracking system (Noldus, Netherlands). Following acclimatization, PND 28 pups were placed in an open-field experimental box to monitor repetitive/stereotypical behaviors. Subsequently, pairs of pups were introduced into the same boxes to observe social interactions. At PND 56, the pups were subjected to the three-chamber sociability test (Fig. 1G).

### Sample collection

At PND 14, 28, 42, and 56, brain tissues were collected from the control and VPA groups (*n* = *4*) to investigate temporal differences. To study the effects of remyelination, brain tissues were harvested from the control, clemastine, VPA, and VPA+clemastine groups (*n* = *6*) at PND 56. Additionally, PND 28 brain tissues, utilized to explore the dysmyelination induced by GDM and prenatal VPA exposure, were obtained from pups in the control, VPA, GDM, and GDM+VPA groups (*n* = *6*). The mice were administered a 50 mg/kg dose of 1% sodium pentobarbital as anesthesia and were then perfused with a 4% paraformaldehyde solution. The brains were extracted, weighed, and left to dehydrate in gradient ethanol overnight before being embedded in paraffin. Based on the anatomical location of the corpus callosum [39], these paraffin-embedded brains were sectioned into 3.5 μm slices.

Brain samples from identical mouse groups were harvested concurrently, employing a consistent methodology (*n* = *6*). The samples underwent dehydration in a graduated sucrose solution overnight before being encapsulated in an optimal cutting temperature compound (SAKURA, YZ4583). Subsequently, the specimens were flash-frozen at −80 °C for 5 min, sectioned into 15.0 μm slices using a freezing microtome (Leica, CM1950, USA), and preserved at −20 °C.

### Chemical staining

Selected cerebral paraffin sections from the control, VPA, GDM, and GDM+VPA groups were utilized for further analysis. These sections were first incubated at 60 °C for 2 h, followed by dewaxing with xylene for 30 min and a successive rehydration through a gradient alcohol procedure. Luxol fast blue (LFB) staining was then implemented, adhering to the methodology outlined by Xie et al. [40]. Imagery was captured at 400× magnification using the fluorescence microscope (Olympus, BX63, Japan).

OLN-93 cells were seeded in 96-well plates at 4 × 10^3^/well. After 12 h of growth, they were divided into eight groups with varying concentrations of GABA (0 mM, 2.5 mM, 5 mM, 10 mM, 25 mM, 50 mM, 100 mM, 250 mM), eight groups with varying concentrations of VPA (0 mM, 0.25 mM, 0.5 mM, 1.0 mM, 2.5 mM, 5.0 mM, 5.0 mM 10.0 mM, 25.0 mM), four groups with varying concentrations of Bicuculline (0 μM, 25 μM, 100 μM, 400 μM), and four groups with varying concentrations of CGP52432 (0 μM, 5 μM, 25 μM, 125 μM). The drugs were administered to each group for 24 h. The Cell Counting Kit-8 (CCK-8) assay was conducted according to standard procedures. Absorbance values indicative of cell activity were measured using the Multiskan^TM^ FC Microplate Photometer (Thermo Fisher Scientific, 1410101, USA).

OLN-93 cells were seeded in 6-well plates at 2 × 10^5^/well and subsequently divided into twelve groups. Six of these groups (blank, GABA, GABA+Bicuculline, GABA+CGP52432, GABA+0.25 mM VPA, and GABA+0.5 mM VPA) were cultured under normal conditions, while the other six groups (HFHG, HFHG+GABA, HFHG+GABA+Bicuculline, HFHG+GABA+CGP52432, HFHG+GABA+0.25 mM VPA, and HFHG+GABA+0.5 mM VPA) were cultivated in a HFHG medium. These groups underwent the same treatment protocol as delineated in the model induction section. Following fixation and crystal violet staining, images were captured from each well at 200× magnification using the fluorescence microscope (Olympus, IXplore, Japan). Additionally, in the 96-well plates, OLN-93 cells were seeded at 4 × 10^3^/well (6 wells per group), and separated into the same 12 groups as described for the crystal violet staining procedure. The previous treatment regimen and CCK-8 assay were utilized to evaluate the cell activity of OLN-93.

### Transmission electron microscopy

On PND 28, pups from the control, VPA, GDM, and GDM+VPA groups (*n* = *4*) were administered 50 mg/kg of sodium pentobarbital for anesthesia. Subsequently, pre-cooled saline was infused, and a 1 × 1× 1 mm section of the corpus callosum was extracted from each brain and immersed in a 2.5% glutaraldehyde solution for 24 h. These sections were then fixed and sectioned into 70 nm slices. Following lead citrate staining, images were acquired at 1200×, 10,000×, and 100,000× magnification using a transmission electron microscope (TEM, Hitachi, H-7500, Japan).

### Immunostaining

Paraffin-embedded cerebral sections from selected pups in the control, clemastine, VPA, and VPA+clemastine groups, as well as in the control, VPA, GDM, and GDM+VPA groups were randomly selected. Following the dewaxing and rehydration protocols outlined in the LFB staining methodology, antigen retrieval was performed using enhanced sodium citrate. Immunohistochemical staining was conducted using the universal mouse/rabbit polymer method detection system (ZSGB Biotech, PV-6000) [41] and the DAB chromogenic kit (ZSGB Biotech, ZLI-9019). Primary antibodies, CNPase (1:400, ab277621) and myelin basic protein (1:800, 78896 s), were diluted with a 3% BSA solution. Following hematoxylin staining and dehydration processes, images were captured at a 400× magnification using a fluorescence microscope (Olympus, BX63, Japan).

Frozen brain slices were randomly selected from the pups in the control, clemastine, VPA, and VPA+clemastine group. Following antigen retrieval and blocking, mixed primary antibodies were applied, including Olig2 (1:200, 65915S, anti-rabbit) and PDGFRα (1:100, 12140181, anti-rat), Olig2 (1:200, anti-rabbit) and CC1 (1:50, OP80, anti-mouse), and PDGFRα (1:100, ab203491, anti-rabbit) and PCNA (1:100, ab29, anti-mouse) were used for 24 h at 4 °C. Subsequently, corresponding secondary antibodies, labeled with Alexa Fluor 647-labeled Goat Anti-Rabbit IgG (1:500, A0468) and Alexa Fluor^TM^ 488 Goat anti-Rabbit IgG (1:500, A11008), Alexa Fluor 647-labeled Goat Anti-Rabbit IgG (1:500) and Alexa Fluor^TM^ 488 Goat anti-Rat IgG (1:500, A11006), and Alexa Fluor Cy3-labeled Goat Anti-Mouse IgG (1:500, A0521) and Alexa Fluor 488^®^ Goat Anti-Rabbit IgG H&L (1:1000, ab150077), were applied in accordance with the primary antibodies used. The sections were covered with antifade mounting medium with DAPI on the slides. In the control, VPA, GDM, and GDM+VPA group, two other sets of mixed antibodies, including p44/42 MAPK (1: 100, 4695 T, anti-rabbit) and NG2 (1:200, mab5384-I, anti-mouse) and phospho-p44/42 MAPK (1:100, 4370 T, anti-rabbit) and NG2 (1:200, anti-mouse), were added. Alexa Fluor Cy3-labeled Goat Anti-Mouse IgG (1:500) and Alexa Fluor 488^®^ Goat Anti-Rabbit IgG H&L (1:1000) were used as the mixed secondary antibodies. All of the pictures were taken at 400× magnification using the laser scanning confocal microscope (Zeiss, LSM880, Germany). Fluorescence was excited at 380 nm (DAPI), 488 nm (488), 550 nm (Cy3), or 647 nm (647) wavelengths.

### Immunoblotting

The total proteins from OLN-93 cells were extracted from the groups described in the crystal violet staining using RIPA lysis buffer, phosphatase inhibitor cocktail A, protease inhibitor cocktail, and 0.1 M EDTA (pH 8.0). Following the method of Liu et al. [42], the nuclear protein extraction kit (Solarbio, R0050) was utilized to extract nuclear proteins from OLN-93 cells. The protein concentration of the samples was determined using a bicinchoninic acid protein assay kit (Beyotime, P0012). After adjusting the concentration, the protein was combined with SDS-PAGE sample loading buffer. Lysates of 10 μl were separated on BeyoGel^TM^ plus precast PAGE gel (Beyotime, P0452) and transferred to 0.2 μm PVDF membranes following standard procedures. The primary antibodies utilized in the western blotting included p44/42 MAPK (1:1000, ab200708), phospho-p44/42 MAPK (1:1000, anti-rabbit), MEK1/2 (1:1000, 8727 T, anti-rabbit), mTOR (1:1000, 2983 T, anti-rabbit), phospho-mTOR (1:1000, 2976S, anti-rabbit), Akt (1:1000, 4685S, anti-rabbit), phospho-Akt (1:1000, 4060 T, anti-rabbit), DUSP-5 (1:1000, ab200708, anti-rabbit), HDAC-1 (1:1000, 34589 T, anti-rabbit), HDAC-2 (1:1000, AF1555, anti-rabbit), HDAC-3 (1:1000, 85057S, anti-rabbit), HDAC-8 (1:1000, AF2737, anti-rabbit), β-actin (1:3000, MA1-140, anti-rabbit), β-tubulin (1:3000, MA5-16308, anti-mouse), and LaminB1 (1:1000, GTX103292, anti-rabbit). Following a secondary antibody incubation, images were captured with the assistance of BeyoECL star kit (Beyotime, P0018AS) and ImageQuant (GE, LAS4000, America).

### Statistical analysis

The results of the behavioural tests were analyzed using Noldus EthoVision-XT 15. ImageJ 2.35 facilitated the analysis of chemical and immunohistochemical staining results, evaluating both the mean and integral optical density values. Additionally, the proportion of double-labeled cell number, area, and intensity derived from the immunofluorescence staining, the cell density of crystal violet staining, and band intensity in western blotting were analyzed using ImageJ. Myelinated axon density, total axon density, myelinated fiber ratio, and g-ratio were evaluated as results of transmission electron microscopy using the software developed by Zaimi et al. [43]. Quantitative data were expressed as mean ± SD and analyzed using ANOVA through SPSS 21.0. Raw data were compiled using Microsoft Excel. Count data were presented as median ± 95% confidence interval and examined with Pearson’s chi-squared test. A significant difference was denoted by *P* < 0.05.

## Results

### Offspring exposed to GDM and prenatal VPA also exhibit social impairments

Female mice with heterozygous leptin receptor deficiencies were administered a 45 kcal% high-fat diet to establish a GDM model incorporating both obesity and genetic factors. And they were administered with VPA on E+12.5d (Fig. 1A). Compared to the control group, the GDM and GDM+VPA groups displayed a progressive increase in fasting blood glucose levels post-pregnancy (Fig. 1B-a), with noticeable differences in body weight emerging from day E+14.5, concurrently with the discernible alterations in fasting blood glucose (Fig. 1B-b). Moreover, these groups exhibited elevated blood glucose levels and a diminished glycemic recovery rate during the OGTT in pregnancy, following a 20% glucose intragastric injection (Fig. 1B-c), affirming the successful establishment of the GDM model. Nonetheless, no significant differences were observed in the offspring’s blood glucose and body weight (Supplementary Fig. 1A, B), highlighting the prenatal influence of GDM’s hyperglycemic factor on progeny. Furthermore, the combination of GDM and prenatal VPA exposure escalated the rates of reproductive failure and abortion/stillbirth significantly compared to either the VPA or GDM groups alone (Fig. 1C-a, b), though postnatal brain development indicators remained unaffected (Fig. 1D, Supplementary Fig. 1C–E).

At PND 28, pups from the GDM+VPA and VPA groups exhibited increased grooming behaviors (Fig. 1E-a) and reduced exploration near the cage walls during the open field test (Fig. 1E-b). Furthermore, they engaged in fewer social interactions, encompassing behaviors such as sniffing, following, and propelling (Fig. 1F-a–F-c). While the GDM group demonstrated similar patterns of interaction deficits and repetitive behaviors, the differences were not statistically significant. Notably, all PND 56 pups maintained their athletic ability (Fig. 1H-b, Fig. 1I-a–I-d) and location preferences (Fig. 1H-a, Fig. 1I-e–I-d) during the initial phase of the three-chamber test. However, during the subsequent stages, the GDM and GDM+VPA groups displayed a pronounced lack of social interest, showcasing a preference for an object over a mouse (Fig. 1H-c–H-d, Fig. 1I-i–I-p), followed by an evident aversion to interaction with a strange mouse in the final stage of the test (Fig. 1H-e–H-f, Fig. 1I-q–I-x). This data underscores that offspring in the GDM+VPA group exhibited parallel social deficiencies when encountering unfamiliar mice, akin to the VPA group, although the GDM group’s social interaction deficit leaned more towards social avoidance. Consequently, the synergistic impact of GDM and prenatal VPA on social interaction mirrored the independent effect of prenatal VPA exposure.

### Demyelination induced by prenatal VPA can be restored by Clemastine

**Fig. 2:**
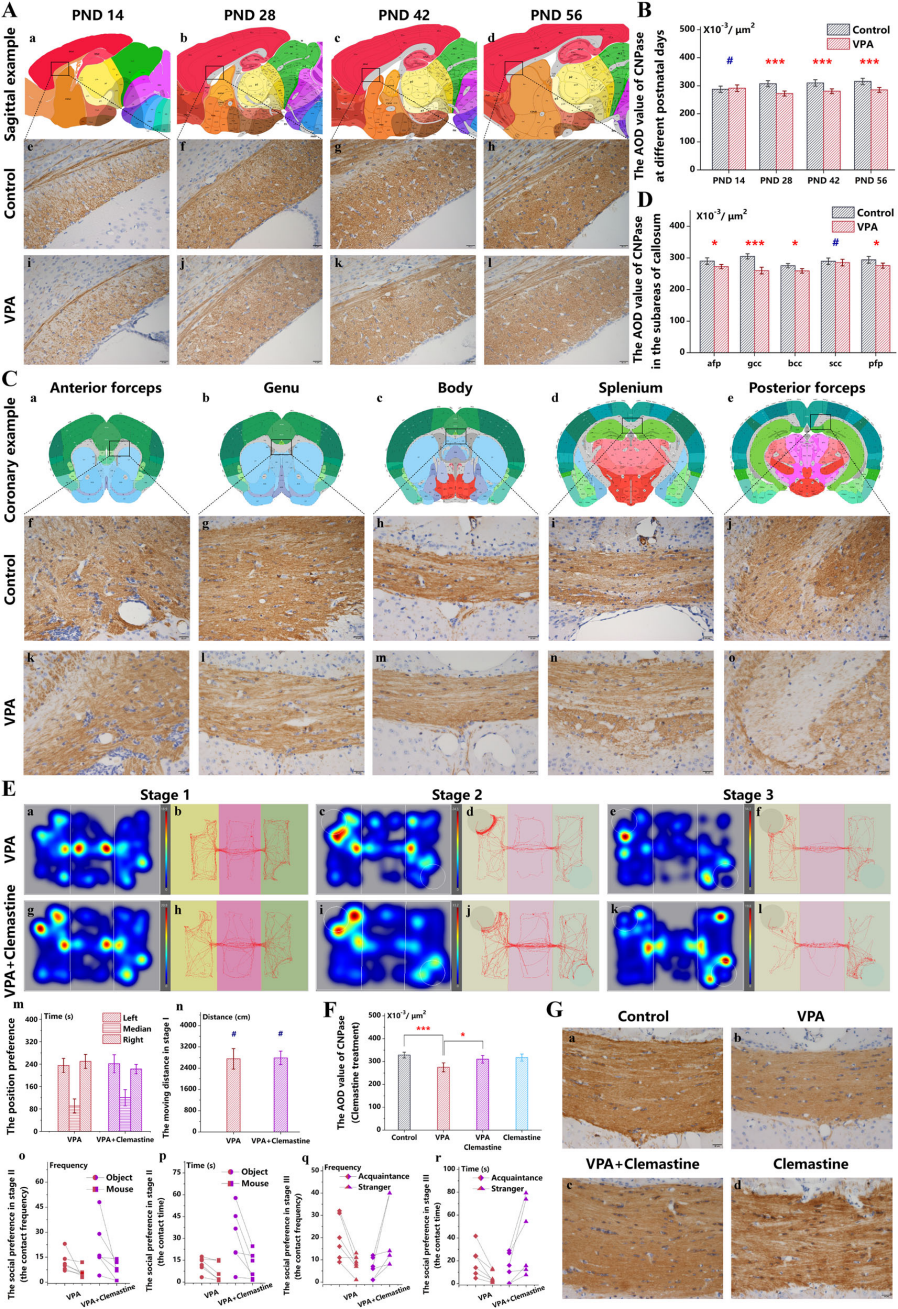
Demyelination induced by prenatal VPA can be restored by Clemastine. **A** At PND 14, 28, 42, and 56, myelin specific CNPase in the CC in the control (**e**–**h**) and VPA (**i**–**l**) groups were exhibited with immunohistochemical staining, based on the sagittal brain disgrams at different postnatal ages (**a**–**d**). **B** The AOD value of immunohistochemical CNPase in the control and VPA groups on different postnatal days (*n* = *4*). **C** Myelin specific CNPase in the control and VPA groups in the anterior forceps **f,** **k**, genu **g,** **l**, body **h,** **m**, splenium **i,** **n**, and posterior forceps **j,** **o** of the CC were exhibited with immunohistochemical staining, based on the coronal brain disgrams of different subregions **a**–**e**. **D** The AOD value of immunohistochemical CNPase in the control and VPA groups in the different subregions of the CC (*n* = *4*). **E** The three-chamber sociability test in the VPA group **a**–**f** and VPA+Clemastine group **g**–**l** at PND 56 (*n* = *6*). The location preferences **m** and athletic ability **n** in the initial stage, the preference for the object over the mouse in the second stages **o,** **p**, and the preference for the strange mouse over the familiar mouse in the third stages **q,** **r** were analyzed as mentioned in Fig. 1. **F** Myelin specific CNPase in the gCC in the control **a**, VPA **b**, VPA+clemastine **c**, and clemastine **d** groups were exhibited with immunohistochemical staining. **G** The AOD value of immunohistochemical CNPase in the control, VPA, VPA+clemastine, and clemastine groups (*n* = *6*). ***P < 0.001, *P < 0.05, compared with two groups. ^#^*P* > 0.05, compared with two groups.

Given the peak period of myelination in the corpus callosum, we introduced two test intervals: 14 days before and after this peak. Analysis of the average optical density (AOD) value of CNPase in the corpus callosum indicated that, at PND 14, the myelination levels in the VPA group pups were not significantly divergent from those in the control group (Fig. 2A-a, e, i, Fig. 2B). However, from PND 28 onwards, a marked difference became evident, continuing until PND 56 (Fig. 2A, B). Early in this stage, the pups displayed characteristic behavioral patterns and diminished social interaction, as noted in earlier descriptions. Notably, in the VPA group, the anterior forceps region of the corpus callosum experienced a substantial reduction in the AOD value of CNPase (Fig. 2A-a, f, k, Fig. 2D), whereas an increase was observed in the subsequent four subregions (Fig. 2C, D). The genu of the corpus callosum (gCC) presented the most pronounced variation (Fig. 2C, D), highlighting its critical role in facilitating inter-hemispheric connectivity and synchronization between both frontal cortices, factors instrumental in social interaction [44]. Consequently, the gCC emerged as a prime focal point for investigating dysmyelination in offspring subject to prenatal VPA exposure, particularly at the PND 28 stage.

From PND 28, a period marked by significant dysmyelination in the gCC induced by prenatal VPA exposure, clemastine was administered intraperitoneally for 21 days, aligning with a myelin sheath formation cycle orchestrated by OLs [45]. This intervention aimed to stimulate remyelination. At PND 56, the VPA+clemastine group exhibited a notable increase in the AOD of CNPase in the gCC compared to the VPA group, albeit it was marginally lower than the control group (Fig. 2F, G). Furthermore, the VPA+clemastine group displayed a restoration in social interactions during the third phase of the three-chamber test, indicating an improvement compared to the VPA group (Fig. 2E-e, f, k, l, p, r). This data reaffirms the correlation delineated by Uccelli et al. [46], associating compromised myelination in the corpus callosum with the manifestations of VPA-induced autism.

### Gestational diabetes and prenatal VPA also lead to demyelination

**Fig. 3:**
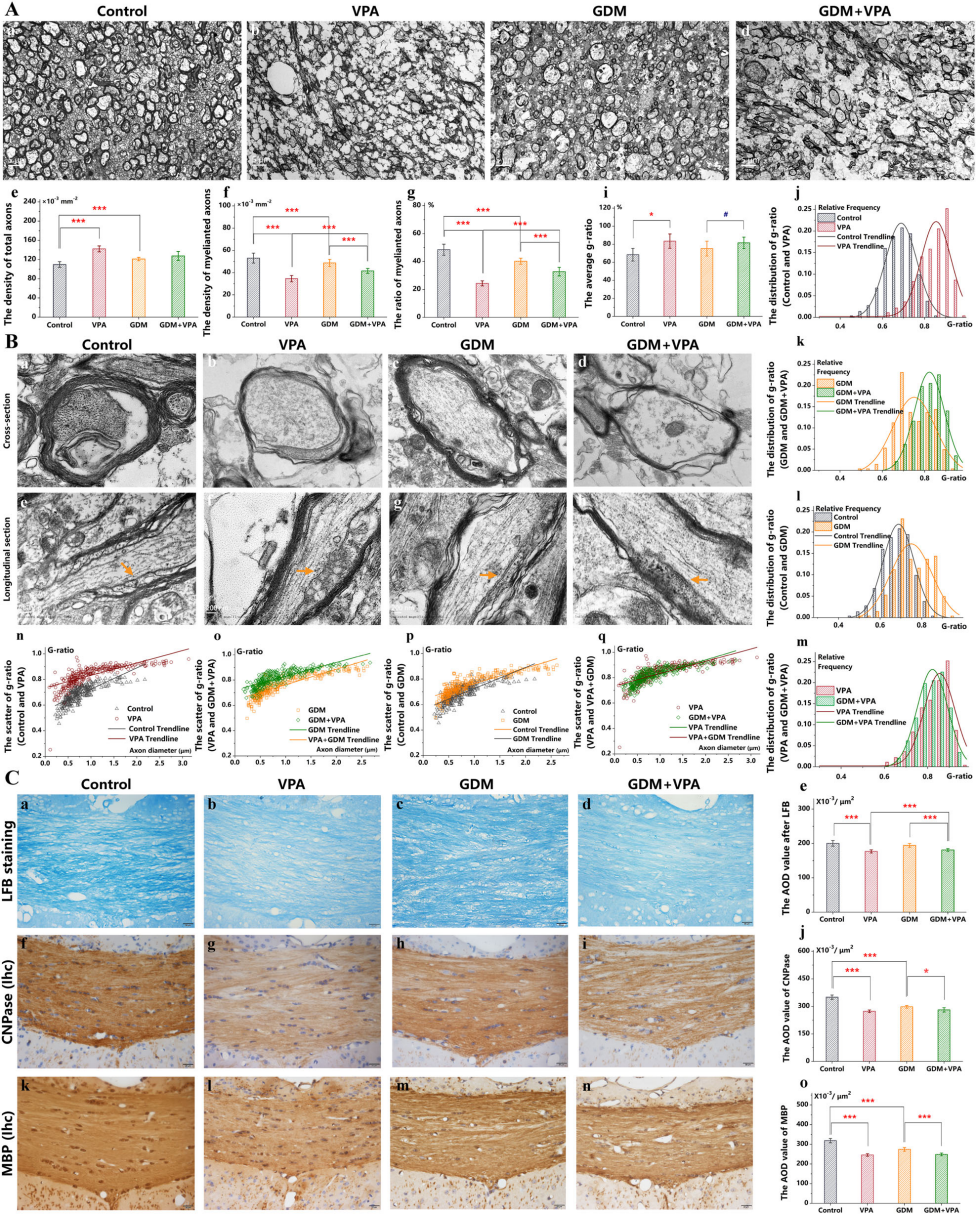
Gestational diabetes and prenatal VPA also lead to demyelination. **A** By PND 28, the proportion of myelinated axons was visualized with TEM at 1000× magnification in the gCC in the control, VPA, GDM, and GDM+VPA groups (**a**–**d**). The total axonal density **e**, the myelinated axonal density **f**, and the proportion of myelinated axons **g** were counted (*n* = *4*). **B** Myelin thickness in the cross sections of axons (**a**–**d**) and the arrangement of paranodes in the longitudinal sections of axons (**e**–**h**) were visualized with TEM at 10,000× and 100,000× magnification in the gCC in these groups (*n* = *4*). The mean g-ratio **i** and the g-ratio distribution (**j**–**m**) and scatter trend (**n**–**q**) were counted to evaluate the myelin thickness. **C** LFB staining (**a**–**d**) and immunohistochemical staining of CNPase (**f**–**i**) and MBP (**k**–**n**) were utilized to exhibit the disrupted myelin lipids and myelin specific proteins in the gCC in the control, VPA, GDM, and GDM+VPA groups (*n* = *6*). The AOD values for both LFB staining **e** and immunohistochemical staining of CNPase **j** and MBP **o** were counted. ***P < 0.001, *P < 0.05, compared with two groups. ^#^*P* > 0.05, compared with two groups.

At PND 28, a time when prenatal VPA engendered notable dysmyelination, the VPA+GDM group displayed a reduced density and proportion of myelinated axons in the gCC compared to the GDM group (Fig. 3A-a–A-g). Analysis of individual axon myelin microstructure revealed an elevated mean g-ratio in the VPA+GDM group (Fig. 3B-a, b, c, d, i), prompting a detailed exploration of the g-ratio distribution and scatter trend [47]. This group exhibited a rightward shift in the frequency distribution of the g-ratio (Fig. 3B-k) and a decreased fitting line slope correlating g-ratio with axon diameter (Fig. 3B-o). In sections parallel to axonal extension direction, the GDM+VPA group manifested a more disrupted paranodal arrangement compared to the GDM group (Fig. 3B-g, h), indicating a more destructed myelin sheath in the GDM+VPA group. Concurrently, diminished AOD values for both LFB staining (Fig. 3C-c–C-e) and immunohistochemical staining of CNPase (Fig. 3C-h–C-j) and MBP (Fig. 3C-m–C-o) were noted in the GDM+VPA group, signifying a heightened impact on myelin-specific proteins and lipids compared to the GDM group. The difference between the GDM+VPA group and the GDM group mirrored the differences between the VPA group and the control group in several aspects, including the density and proportion of myelinated axons (Fig. 3A-a–A-g), the mean and distribution of the g-ratio (Fig. 3B-i–C-m), the disordered arrangement of paranodes (Fig. 3B-e, h), and the decreased levels of myelin-specific proteins (Fig. 3C-f–C-o) and myelin lipids (Fig. 3C-a–C-e). Compared to the control pups, those in the GDM group exhibited a significant decrease in myelin-specific proteins (Fig. 3C-f, h, j, k, m, o) and a tendency to have reduced numbers of myelinated axons (Fig. 3A-a, c, g) and myelin lipids (Fig. 3C-a, c, e). This suggests that two distinct factors, GDM and prenatal VPA, both contribute to dysmyelination in the gCC. However, the alterations in dysmyelination from the GDM+VPA group to the GDM group were less pronounced than those from the VPA group to the control group. It seemed that two interfering factors in the GDM+VPA group had opposite effects. Therefore, the dysmyelination in the offspring induced by concurrent prenatal VPA and GDM exposure was comparable to that caused by prenatal VPA alone. Given that GDM predisposes to dysmyelination, the combination of GDM and prenatal VPA appeared to exert a contrasting effect on myelination.

### Insufficient differentiation of OPC cells associated with demyelination

**Fig. 4:**
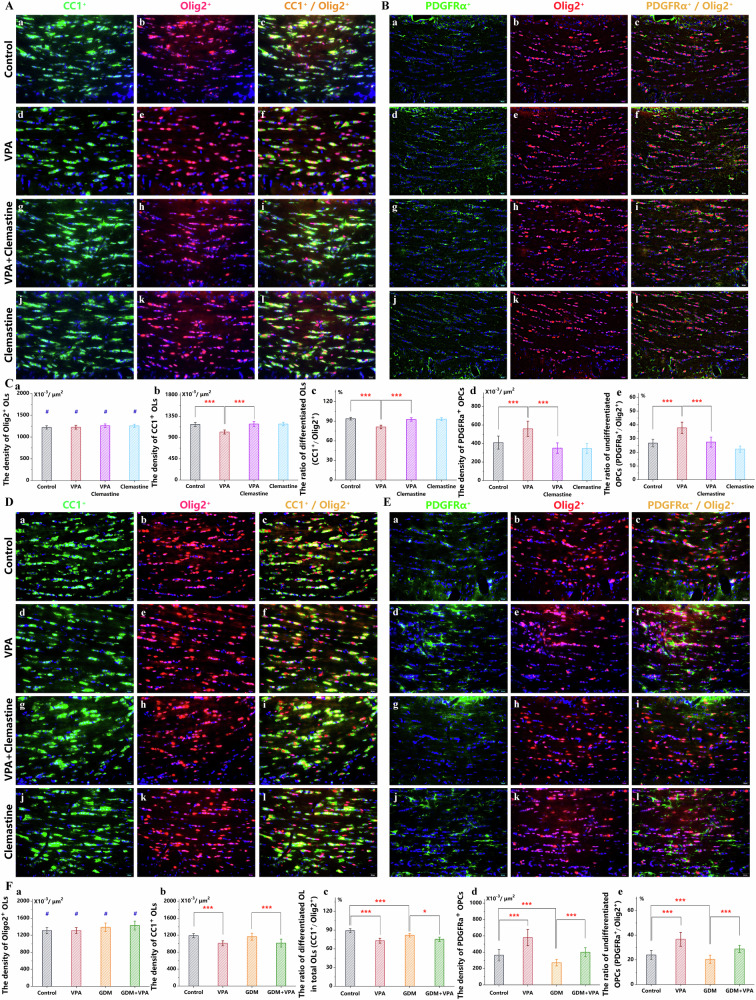
Insufficient differentiation of OPC cells associated with demyelination. **A** Immunofluorescence staining for CC1 (green) displayed mature-OL **a,** **d,** **g,** **j** and for Olig2 (red) displayed oligodendrocyte lineage cells **b,** **e,** **h,** **k** in the gCC in the control **c**, VPA **f**, VPA+clemastine **i**, and clemastine **l** groups (*n* = *6*) at PND 56. DAPI (blue) was used as a nuclear counterstain. **B** Immunofluorescence staining for PDGFRα (green) displayed OPCs **a,** **d,** **g,** **j** and for Olig2 (red) displayed oligodendrocyte lineage cells **b,** **e,** **h,** **k** in the gCC in the control **c**, VPA **f**, VPA+clemastine **i**, and clemastine **l** groups (*n* = *6*) at PND 56. DAPI (blue) was used as a nuclear counterstain. **C** The Olig2^+^ OLs density **a**, the CC1^+^ mature-OL density **b**, the proportion of CC1^+^ differentiated OLs in Olig2^+^ total OLs **c**, the PDGFRα^+^ OPCs density **d**, and the proportion of PDGFRα^+^ OPCs in Olig2^+^ total OLs **e** were counted. **D** Immunofluorescence staining for CC1 (green) displayed mature-OL **a,** **d,** **g,** **j** and for Olig2 (red) displayed oligodendrocyte lineage cells **b,** **e,** **h,** **k** in the gCC in the control **c**, VPA **f**, GDM **i**, and GDM+VPA **l** groups (*n* = *6*) at PND 28. DAPI (blue) was used as a nuclear counterstain. **E** Immunofluorescence staining for PDGFRα (green) displayed OPCs **a,** **d,** **g,** **j** and for Olig2 (red) displayed oligodendrocyte lineage cells **b,** **e,** **h,** **k** in the gCC in the control **c**, VPA **f**, GDM **i**, and GDM+VPA **l** groups (*n* = *6*) at PND 28. DAPI (blue) was used as a nuclear counterstain. **F** The Olig2^+^ OLs density **a**, the CC1^+^ mature-OL density **b**, the proportion of CC1^+^ differentiated OLs in Olig2^+^ total OLs **c**, the PDGFRα^+^ OPCs density **d**, and the proportion of PDGFRα^+^ OPCs in Olig2^+^ total OLs **e** were counted. ***P < 0.001, *P < 0.05, compared with two groups. ^#^*P* > 0.05, compared with two groups.

At PND 56, after a 21-day intraperitoneal clemastine regimen, the VPA+clemastine group exhibited fewer undifferentiated OPCs than the VPA group (Fig. 4B-a, d, g, j, Fig. 4C-d). Despite consistent Olig2^+^ positive oligodendroglial lineage cell density (Fig. 4A-b, e, h, k, Fig. 4B-b, e, h, k, Fig. 4C-a), the proportion and density of PDGFRα^+^/Olig2^+^ double-labelled undifferentiated OPCs displayed analogous trends (Fig. 4B-c, f, i, l, Fig. 4C-e). The VPA+clemastine cohort showed elevated density and proportions of differentiated mature-OL with CC1^+^/Olig2^+^ double-labeling compared to the VPA group (Fig. 4A, Fig. 4C-b, c). Notably, differences among the VPA+clemastine, clemastine, and control groups were negligible. This underscores clemastine’s role in enhancing OPC differentiation and maturation, concurrently ameliorating autistic phenotypes and corpus callosum dysmyelination in offspring exposed to prenatal VPA. Therefore, OPC differentiation deficits contribute to VPA-induced dysmyelination.

In the GDM+VPA group, the density of undifferentiated OPCs with PDGFRα^+^/Olig2^+^ double-labeling in the gCC was notably elevated compared to the GDM cohort (Fig. 4E-a, d, g, j, Fig. 4F-d). While the density of Olig2^+^ positive oligodendroglial lineage cells remained stable (Fig. 4E-b, e, h, k, Fig. 4E-c, f, i, l, Fig. 4F-a, e), the proportion of undifferentiated OPCs in the GDM+VPA group increased in line with density. In terms of OPC differentiation shifts, the GDM+VPA group demonstrated reduced density and proportions of differentiated matural-OLs with CC1^+^/Olig2^+^ double-labeling relative to the GDM group (Fig. 4D-a, d, g, j, Fig. 4D-c, f, i, l, Fig. 4F-b, c). When comparing the increased undifferentiation and diminished differentiation, disparities between the VPA and control groups paralleled those between the GDM+VPA and GDM groups. This suggests that VPA, when paired with GDM, induces a deficiency in OPC differentiation akin to the effects of VPA alone. However, the variance from the GDM+VPA to the GDM group was milder than that from the VPA to the control group, indicating GDM’s distinct influence compared to VPA in the GDM+VPA combination. Although gestational hyperglycemia is an isolated dysmyelination factor, there existed only two distinctions between the GDM and control groups: a notable decline in differentiated OL proportions and a downward trend in undifferentiated OPC proportions (Fig. 4F-c, e). This infers differential OPC differentiation impacts of combined GDM and VPA versus GDM in isolation, mirroring their varied influences on dysmyelination.

### Excessive proliferation of OPC cells attributed to the inhibition of GABA receptors

**Fig. 5:**
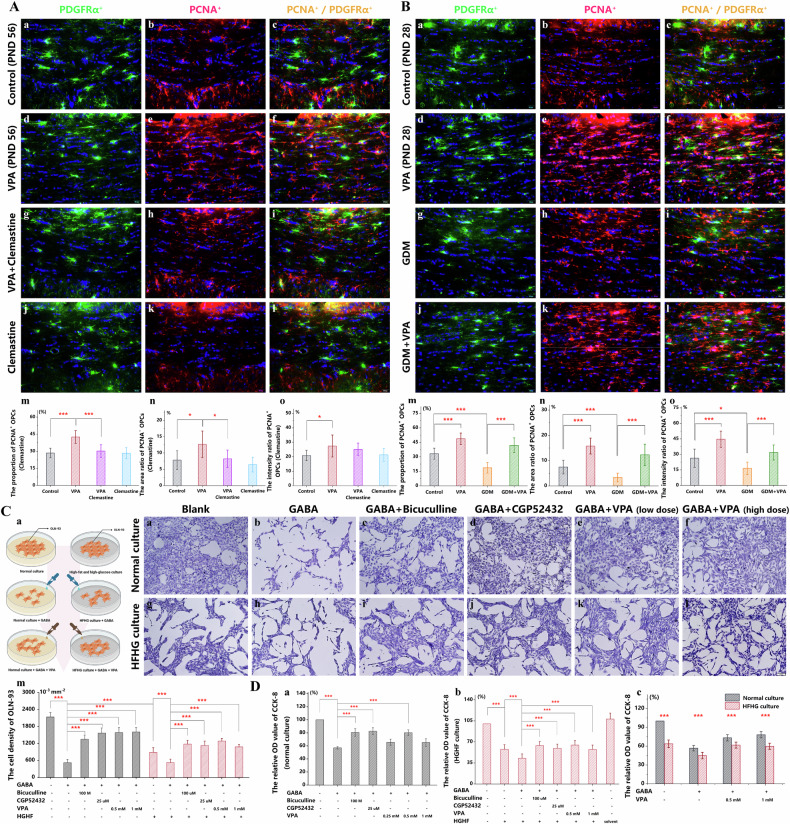
Excessive proliferation of OPC cells attributed to the inhibition of GABA receptors. **A** Immunofluorescence staining for PDGFRα (green) displayed OPCs **a,** **d,** **g,** **j** and for PCNA (red) displayed a proliferation marker **b,** **e,** **h,** **k** in the gCC in the control **c**, VPA **f**, VPA+clemastine **i**, and clemastine **l** groups at PND 56 (*n* = *6*). DAPI (blue) was used as a nuclear counterstain. The proportion **m**, the area ratio **n**, and the intensity ratio **o** of proliferative OPCs (PCNA^+^/ PDGFRα^+^) in PDGFRα^+^ total OPCs were counted. **B** Immunofluorescence staining for PDGFRα (green) displayed OPCs **a,** **d,** **g,** **j** and for PCNA (red) displayed a proliferation marker **b,** **e,** **h,** **k** in the gCC in the control **c**, VPA **f**, GDM **i**, and GDM+VPA **l** groups at PND 28 (*n* = *6*). DAPI (blue) was used as a nuclear counterstain. The proportion **m**, the area ratio **n**, and the intensity ratio **o** of proliferative OPCs (PCNA^+^/ PDGFRα^+^) in PDGFRα^+^ total OPCs were counted. **C** The diagram of the cell models established by GABA and VPA treatment in OLN-93 in the normal culture and the HFHG culture. **D** The crystal violet staining of the OLN-93 treated with with blank, GABA, GABA+Bicuculline, GABA+CGP52432, GABA+VPA (low dose), and GABA+VPA (high dose) in the normal culture (**a**–**f**) and the HFHG culture (**g**–**l**) (*n* = *4*). The cell density for each group was counted **m**. ***P < 0.001, *P < 0.05, compared with two groups.

At PND 56, an analysis was conducted following a 21-day intraperitoneal administration of clemastine, assessing OPC proliferation through the comparison of cell quantity ratios, dual-labeled area, and fluorescence intensity between proliferative (PCNA^+^/ PDGFRα^+^) and total OPCs. Results indicated a notable decline in the cell quantity and fluorescence intensity ratios in the VPA+clemastine group compared to the VPA group (Fig. 5A-c, f, i, l, m, n), accompanied by a diminished trend in the dual-labeled area (Fig. 5A-o). These findings signify that prenatal VPA exposure is an underlying factor contributing to dysmyelination, linked to a proliferation deficit in OPCs. Similarly, alterations in OPC proliferation in the GDM+VPA group mirrored those observed in the VPA group, albeit with a less pronounced effect when juxtaposed with the impacts of prenatal VPA alone. Notably, the variations in the aforementioned metrics between the GDM+VPA and GDM groups were less stark than those between the VPA and control groups (Fig. 5B- i, l, m, n, o). Consequently, the GDM group exhibited a reduction in OPC proliferation as delineated by the aforementioned parameters (Fig. 5B-c, f, m, n, o), inferring that the collective influence of prenatal VPA and GDM on OPC proliferation parallels their independent actions.

The high fat-high glucose (HFHG) is implicated as a principal agent in offspring damage resulting from GDM [48, 49]. At the operable concentrations verified through CCK-8 (Fig. 5C, Supplementary Fig. 2A–C), preliminary results underscore that in a GABA-preconditioned normal culture environment, VPA concentrations of 0.5 and 1.0 mM fostered an uptick in cell activity and density, as evidenced by CCK-8 and crystal violet staining, respectively (Fig. 5D-a–f, n). These increments, albeit to a diminished extent, were mirrored in a high-fat and high-glucose milieu preconditioned with GABA (Fig. 5D-g to D-n), paralleling the outcomes elicited by antagonism of GABA_A_ or GABA_B_ receptors. These in vitro findings resonate with in vivo observations, highlighting the antagonistic effects of a high sugar-high fat environment and VPA on the activity and proliferative potential of OLN-93 cells.

### Proliferation-related changes in ERK and its downstream molecules in OPCs

**Fig. 6:**
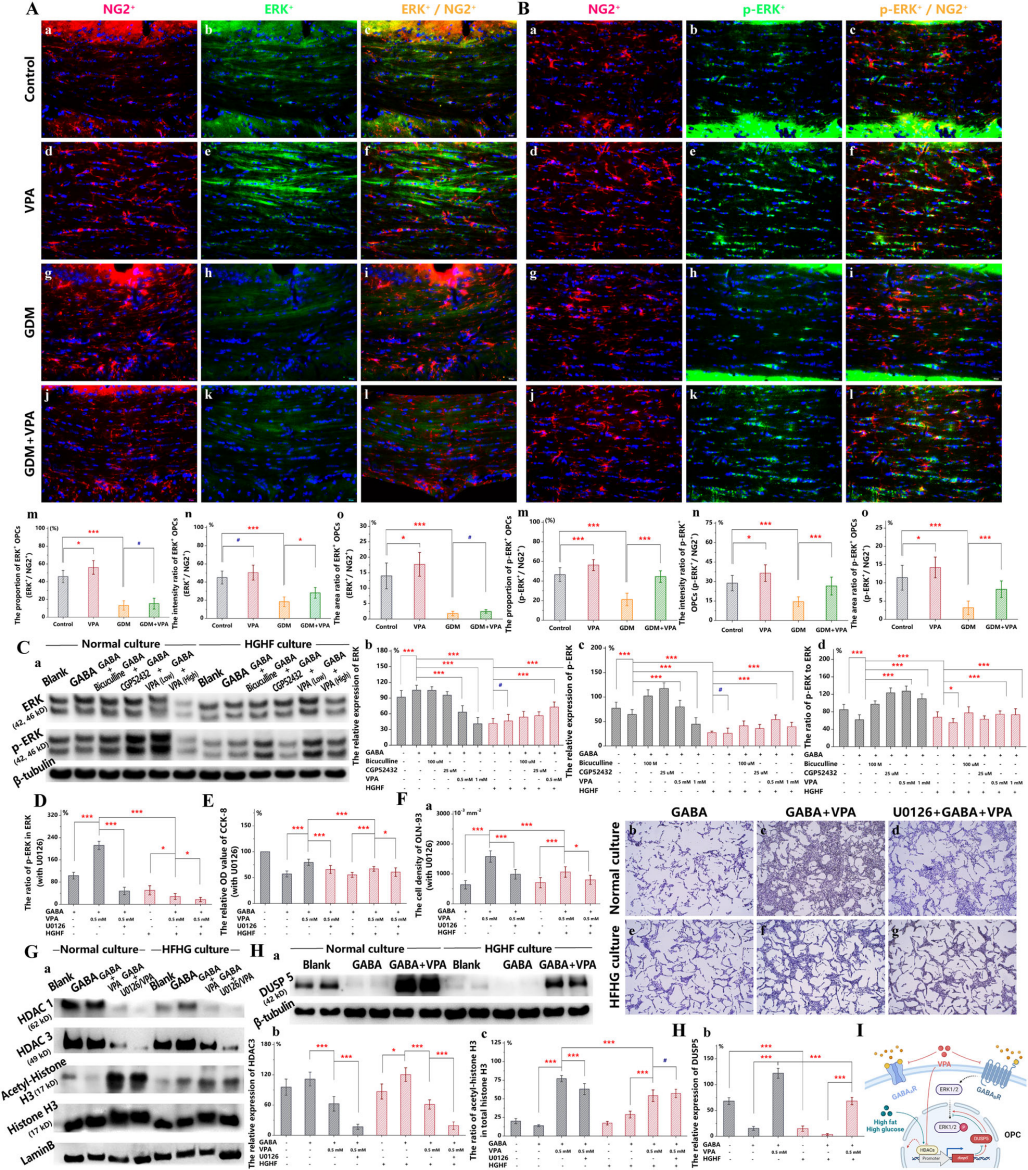
Proliferation-related changes in ERK and its downstream molecules in OPCs. **A** Immunofluorescence staining for ERK (green) **a,** **d,** **g,** **j**) and for NG2 (red) displayed OPCs **b,** **e,** **h,** **k** in the gCC in the control **c**, VPA **f**, GDM **i**, and GDM+VPA **l** groups at PND 28 (*n* = *6*). DAPI (blue) was used as a nuclear counterstain. The proportion **m**, the area ratio **n**, and the intensity ratio **o** of ERK^+^ OPCs in total OPCs were counted. **B** Immunofluorescence staining for p-ERK (green) **a,** **d,** **g,** **j** and for NG2 (red) displayed a OPCs **b,** **e,** **h,** **k** in the gCC in the control **c**, VPA **f**, GDM **i**, and GDM+VPA **l** groups at PND 28 (*n* = *6*). DAPI (blue) was used as a nuclear counterstain. The proportion **m**, the area ratio **n**, and the intensity ratio **o** of p-ERK^+^ OPCs in total OPCs were counted. **C** Expression of ERK and p-ERK in the OLN-93 treated with blank, GABA, GABA+Bicuculline, GABA+CGP52432, GABA+VPA (low dose), and GABA+VPA (high dose) was evaluated by Western blotting **a** (*n* = *4*). β-tubulin expression was served as an internal control. The expression of ERK and p-ERK **b,** **c** and the ratio of p-ERK to ERK **d** were counted. **D** The ratio of p-ERK to ERK in the OLN-93 pretreated with 10 μM U0126 in the normal culture and the HFHG culture. **E** The relative optical density (OD) value of CCK-8 test in the OLN-93 treated with GABA, GABA+VPA, and U0126+GABA+VPA (*n* = *6*). **F** The crystal violet staining of the OLN-93 treated with the methods described in **E** in the normal culture (**b**–**d**) and the HFHG culture (**e**–**g**) (*n* = *4*). The cell density for each group was counted **a**. **G** Expression of HDAC1, HDAC3, histone H3, and acetyl-histone H3 in the OLN-93 treated with blank, GABA, GABA+VPA, and GABA+VPA+U0126 was evaluated by Western blotting **a** (*n* = *4*). LaminB expression was served as an internal control. The expression of HDAC3 **b** and the ratio of acetyl-histone H3 to histone H3 **c** were counted. **H** Expression of DUSP5 in the OLN-93 treated with blank, GABA, and GABA+VPA was evaluated by Western blotting **a** (*n* = *4*). β-tubulin expression was served as an internal control. The expression of DUSP5 were counted **b**. **I** The diagram of intracellular effects of VPA and HFHG in OPCs. ***P < 0.001, *P < 0.05, compared with two groups. ^#^*P* > 0.05, compared with two groups.

In comparison to the control group, the VPA group exhibited a significant increase in the ratios of cell quantity and double-labelled area between ERK^+^/NG2^+^ double-labeled OPC and NG2^+^-labeled total OPC at PND 28 (Fig. 6A-a–A-f, A-m, o). The fluorescence intensity also tended to increase (Fig. 6A-n). Notably, there were minimal OPCs with ERK^+^/NG2^+^ double labeling in the GDM and GDM+VPA groups, thereby illustrating a negligible difference (Fig. 6A-g–A-l, A-m, o). The activation of ERK1/2 was amplified by the phosphorylation at Thr202-Tyr204 and Thr185-Tyr187 of ERK1/2 [50]. Compared with the control group, the VPA group exhibited a significant increase in the ratios of cell quantity, double-labeled area, and fluorescence intensity between p-ERK^+^/NG2^+^ double-labeled OPCs and NG2+-labeled total OPCs (Fig. 6B-a–B-f, B-m, o). The differences observed between the GDM+VPA group and the GDM group were less pronounced than those between the VPA group and the control group (Fig. 6B). Furthermore, the GDM and GDM+VPA groups, characterized by gestational hyperglycemia, demonstrated a notable decline in the quantity of OPCs with p-ERK+/NG2+ double labeling when compared to the group without gestational hyperglycemia (Fig. 6B-g–B-l, B-m, o).

In vitro, ERK expression was higher in the normal culture than in the HFHG culture, although there were no significant differences in the groups of each culture (Fig. 6C-a, b). After 100 mM GABA treatment, the expression of p-ERK in normal culture increased with the addition of 0.5 mM VPA or CGP52432 compared to treatment with GABA alone (Fig. 6C-a, c). In the HFHG culture, the expression of p-ERK rose with 0.5 mM VPA, 1.0 mM VPA or (+)-Bicuculline following 100 mM GABA treatment compared to that with GABA alone, while p-ERK expression was not as high as that in normal culture (Fig. 6C-a, c). The change of ERK phosphorylation ratio was almost consistent with the change of p-ERK expression (Fig. 6C-d). The MAPK signaling pathway in neurons, astrocytes, and microglia, when inhibited by the in vivo injection of U0126, impacted OPCs variably [51]. Moreover, OPC differentiation induced by the ERK inhibitor could not restore processes in chronic demyelination [52]. According to Singh et al. [53], 10 μM U0126 inhibited ERK in OLN-93 cells. ERK and p-ERK expressions in VPA-affected groups significantly decreased after U0126 pretreatment in both HFHG and normal cultures (Supplementary Fig. 3B, Fig. 6D). When ERK inhibition occurred, both the increased cell viability (Fig. 6F) and proliferation (Fig. 6E) caused by GABA-pretreated VPA returned to the levels observed with GABA alone. There was no significant difference between the groups in the HFHG culture and those in the normal culture. It appears that ERK activation in the HFHG culture could not be further inhibited by U0126 due to the suppressive effects of high-fat and high-glucose environments.

As an inhibitor of histone acetylase inhibitor (HDACi), VPA exhibited the most significant inhibitions in the levels of histone acetylase 3 (HDAC3) among the class I HDACs in the OPCs (Supplementary Fig. 3C). Consequently, the impact of VPA and high-fat, high-glucose environments on the expression levels of HDAC3 and DUSP5 in OLN-93 cells was investigated in vitro. Comparing the control and GABA groups in the HFHG culture with those in the normal culture, there was no significant difference in HDAC3 expression, although a rising trend was noted in the HFHG culture (Fig. 6G-a, b). Pretreatment with GABA followed by VPA led to a reduction in HDAC3 expression in both the HFHG and normal cultures compared to the GABA group alone (Fig. 6G-a, b). In comparison to the normal culture, VPA treatment resulted in a decrease in the acetylation level of histone H3 at Lys56 in the HFHG culture (Fig. 6G-a, c). It indicated that the high-fat and high-glucose environment caused a decrease in H3K56ac, an increase in H3 deacetylation, and an enhanced activity of HDACs in OPCs. Upon inhibiting ERK with U0126, the expression of HDAC3 further declined in the VPA group pre-treated with GABA in both culture (Fig. 6G-a, b). Compared the GABA group, DUSP5 expression was significantly was elevated in the VPA group pre-treated with GABA in both culture (Fig. 6H-a, b). Furthermore, DUSP5 expression was considerably lower in the HFHG culture than in the normal culture (Fig. 6H-a, b). It appears that the antithetical alterations induced by VPA and HFHG environments in OLN-93 cells, pertaining to the expressions of HDAC3 and DUSP5, correspond with the inverse changes observed in the phosphorylation and activation of ERK, OPC proliferation and differentiation, and the myelination disruptions caused by GDM and prenatal VPA exposure (Fig. 6I).

## Discussion

During embryogenesis, radial glial cells situated in the ventricular zone undergo asymmetric division to form OPCs, which subsequently express specific markers such as NG2 and PDGFRα [45]. Commencing on the ninth embryonic day, successive waves of OPCs originate from various brain regions, reliant on nourishment from astrocytes and axons to flourish and occupy the fetal cerebral space [54–56]. Post-birth, a significant fraction of prenatal OPCs metamorphoses into a distinct subset, maintaining sustained proliferative activity and holding the potential to mature into OLs under particular conditions, thereby facilitating myelin production [57]. The residual prenatal OPCs undertake extensive migration, aligning alongside neuronal cell axons where they differentiate into mature-OLs, contributing to the formation of myelin sheaths [58]. A conducive uterine environment is pivotal for fostering prenatal oligodendroglial genesis and ensuring optimal fetal brain functionality post-birth. Prenatal malnutrition adversely affects various OL-specific molecules during embryonic development, potentially leading to dysmyelination in the corpus callosum and heightened propensity for anxiety-like behavior during adolescence [59]. Analogous repercussions on oligodendroglial maturation, myelination disruption, and postnatal behavioral abnormalities have been reported with exposure to other prenatal factors, including elevated NO_2_ [60], airborne particulate matter [61], fetal ischemia-hypoxia [62], and progesterone insufficiency [63]. Moreover, the offspring’s social deficits and repetitive/stereotypic behaviors, induced by the synergy of GDM and prenatal VPA exposure, coincide with inadequate myelination and an increase in immature OPC presence in the gCC. Overall, this uptick in immature cell populations is attributed to hindered differentiation coupled with amplified proliferation. In the offspring exposed to both GDM and prenatal VPA, the reduced OPC differentiation suggests that these prenatal elements disrupt the native capabilities of prenatal OPCs, which under normal circumstances, would differentiate into mature-OLs and facilitate myelin sheath formation post-birth. The accentuated OPC proliferation indicates that these factors influence a specific OPC subset characterized by persistent proliferative potential. Furthermore, GABA, interacting with both ion channel-linked GABA_A_ receptors and G protein-coupled GABA_B_ receptors found on OPC surfaces, induces diminished OPC proliferation and disrupted myelination [54, 64]. This interaction facilitates the establishment of synapses between OPCs and GABAergic neurons, modulating both OPC proliferation and differentiation [45, 64]. Our findings demonstrate that VPA augments OPC proliferation both in vivo and in vitro by obstructing GABA receptors, whereas GDM and HFHG conditions mimic GABA’s effects in reducing proliferation.

In OPCs, the GABA_B_ modulates a range of intracellular signaling molecules, including Akt/mTOR and ERK through the coupled G protein [65], while the cytosolic Ca^2+^ influx is altered by the GABA_A_ receptor [66]. ERK1/2 is an ubiquitous intracellular signaling molecule that, after phosphorylation, enters the nucleus to regulate gene expression related to cell proliferation, differentiation, and survival [67]. The ERK signaling pathway in oligodendroglial lineage cells governs the proliferation, differentiation, and migration of OPCs and the myelination of mature-OLs [68]. Elevated ERK1/2 activity in Olig1^+^ oligodendroglial lineage cells notably boosts the proliferative capacity and cell number of OPCs [69]. In poorly differentiated cells in the CNS, VPA specifically and sustainably activates ERK phosphorylation, impacting transcription factors such as activator protein-1, cyclic-AMP response binding protein, and peroxisome proliferator-activated receptor γ via pathways independent of c-Jun N-terminal kinase and p38 mitogen-activated protein kinase downstream of ERK [70]. In our research, OPCs displaying precursor cell characteristics in the CNS demonstrated heightened proliferative capacity and cell density due to ERK phosphorylation activation in response to VPA, both in vitro and in vivo. When ERK1/2 is knocked down in Olig2^+^ oligodendroglial lineage cells, OPC proliferation diminishes [71]. Echoing findings from Yang et al. in hippocampal neurons [72], OPCs in a high fat-high glucose environment display reduced ERK phosphorylation, proliferative capability, and cell viability. Ruiz-Palacios et al. [73] also found a decline in OPC proliferative capacity in pups exposed to GDM in vivo. Aligned with these OPC proliferation shifts, pups subjected to gestational hyperglycemia exhibit a marked drop in ERK phosphorylation, irrespective of prenatal VPA presence. The ERK signaling pathway remains vital for OPC differentiation both in vitro and in vivo [74]. In typical and diabetic pregnancies, prenatal VPA augments ERK phosphorylation and activation in OPCs, yet this augmentative effect diminishes when GDM and prenatal VPA coexist. Although our research indicates that prenatal VPA reduces OPC differentiation while boosting ERK activation, this contrasts with prior studies that identified enhanced OPC differentiation following ERK activation [75]. OPC differentiation is governed by multiple intracellular signaling molecules, encompassing mTOR and Akt, in addition to the ERK signaling pathway [76, 77]. As enhancers of OPC differentiation, Akt and mTOR phosphorylation are suppressed by VPA in vitro (Supplementary Fig. 3A). The diminished differentiation observed in OPCs in vivo might stem from the inhibitory influence of Akt and mTOR surpassing the activating impact of ERK.

The DUSP family serves as the primary regulators of the MAPK signaling pathway, influencing its function, signal intensity, and longevity. In various mental illness models, including depression and bipolar disorder, DUSPs modulate the ERK molecule, downstream of the cannabinoid receptor in microglia [78]. Specific DUSP subfamilies feature MAP kinase-binding or kinase-interacting motifs, interacting with MAPK co-docking domains to dictate enzyme-substrate interactions [79]. DUSP5, a characteristic member of the DUSP family, harbors an interacting motif and demonstrates phosphatase activity directed towards ERK [80]. Overexpression of DUSP5 triggers the dephosphorylation and subsequent inactivation of ERK [81]. In our research, we observed that VPA represses HDAC3, fostering DUSP5 promotion alongside ERK activation in oligodendrocyte precursor cells (OPCs), corroborating Habibian et al.’s findings [82]. According to them, VPA functions as an HDAC inhibitor, suppressing HDAC3 expression in OPCs, thereby facilitating histone acetylation which unveils the DUSP5 gene promoter. Consequently, DUSP5 expression is escalated, culminating in augmented ERK phosphorylation and activation. Conversely, the degradation of DUSP5 amplifies both the intensity and persistence of the ERK signal [83]. In the context of a high-fat, high-glucose environment in OPCs, HDAC3 activation is noted, alongside inhibited DUSP5 expression, diminished p-ERK levels, and sustained dephosphorylated ERK, a pattern substantiated by Xu et al. in their research on diabetic encephalopathy [84]. Furthermore, DUSP5 is a transient, ubiquitin-targeted protein for degradation. Notably, ERK fosters DUSP5 stability through ERK-DUSP5 interaction, independent of ERK kinase activity, establishing a form of feedback mechanism [85]. Hence, reduced ERK expression facilitates rapid DUSP5 degradation via ubiquitination, elucidating the observed decrease in DUSP5 expression when ERK was inhibited with U0126 in our study. Although existing literature primarily addresses DUSP1-dependent intracellular transcription factor inactivation in Müller glia, the comparative genesis of OPCs in the corpus callosum and Müller glia in the retina from the subventricular zone suggests the potential for a novel research trajectory focusing on DUSP involvement in OPC myelination.

## Conclusion

In conclusion, prenatal VPA exposure due to GDM results in stereotyped behavior and social interaction deficits in offspring, mirroring the effects of prenatal VPA exposure alone. Although GDM has been proven to impede myelination in the corpus callosum, the dysmyelination from the combined influence of GDM and prenatal VPA is less pronounced than that induced by prenatal VPA in isolation. OPCs originating from the embryonic stage provide a compelling explanation for this dysmyelination, as prenatal insults do not coincide with postnatal myelination. When both factors co-exist, prenatal VPA promotes an increase in immature OPCs through diminished differentiation and enhanced proliferation; in contrast, GDM exerts the inverse effect. Alterations in OPC proliferation are linked to intracellular shifts in ERK phosphorylation both in vitro and in vivo. The activation of DUSP5 combined with the inhibition of HDAC3 induces a rise in ERK phosphorylation, accounting for the VPA-driven impact on OPCs. GDM’s influence on HDAC3 and DUSP5 contrasts with that of VPA, leading to inverse modifications in OPC proliferation and ERK activation.

## Supplementary information

Supplementary Figure 1

Supplementary Figure 2

Supplementary Figure 3

Supplementary Figure Legends and Research Elements
