# Supplementary figures and images for: Prenatal valproic acid on the basis of gestational diabetes also induces autistic behavior and disrupts myelination and oligodendroglial maturation slightly in offspring
Source: Transl Psychiatry. 2025 Aug 7;15:271. doi: 10.1038/s41398-025-03450-z (PMC12332004; doi:10.1038/s41398-025-03450-z)

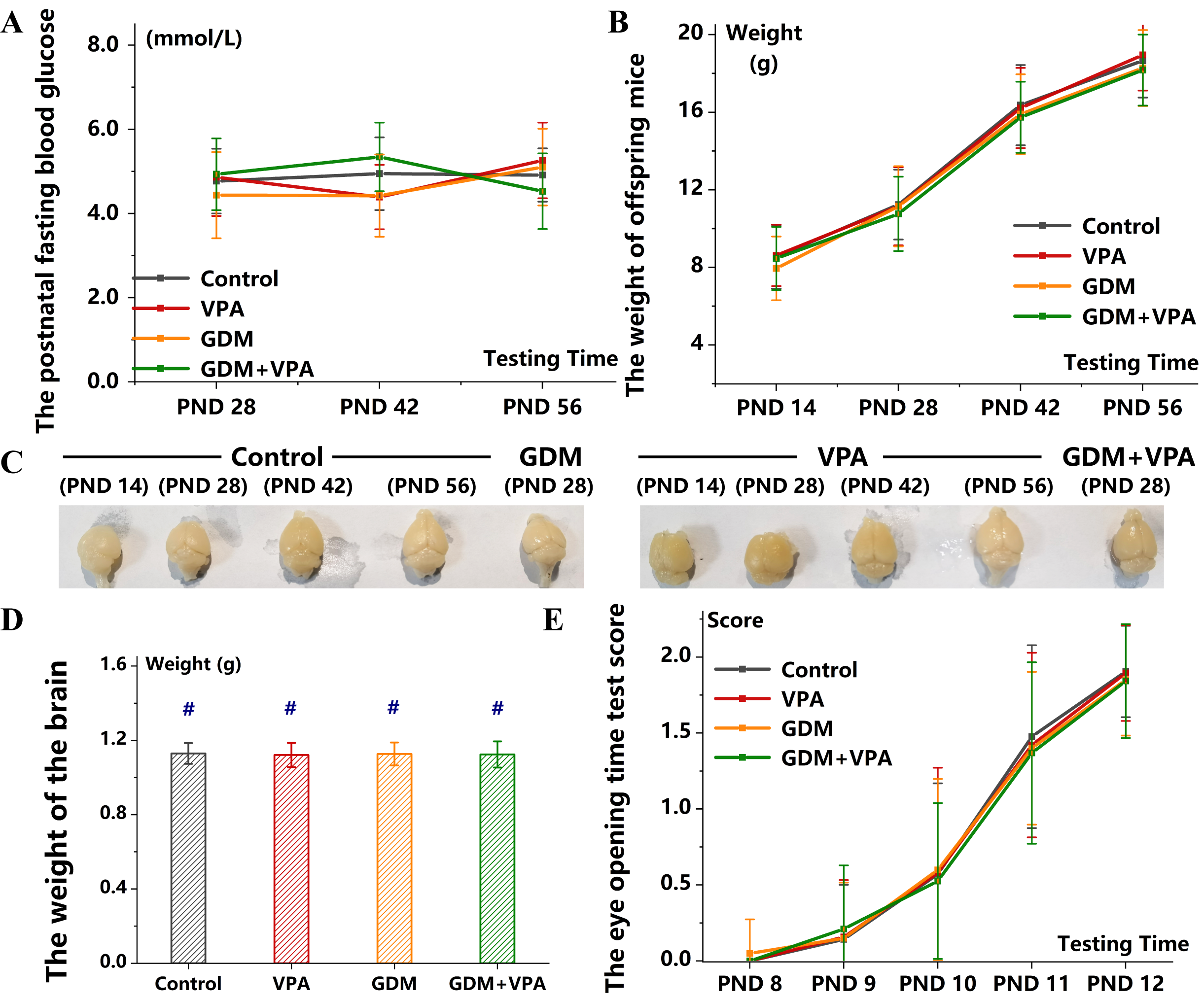

Supplement: Supplementary file 1 — Supplementary Figure 1 [file 41398_2025_3450_MOESM1_ESM.tif]

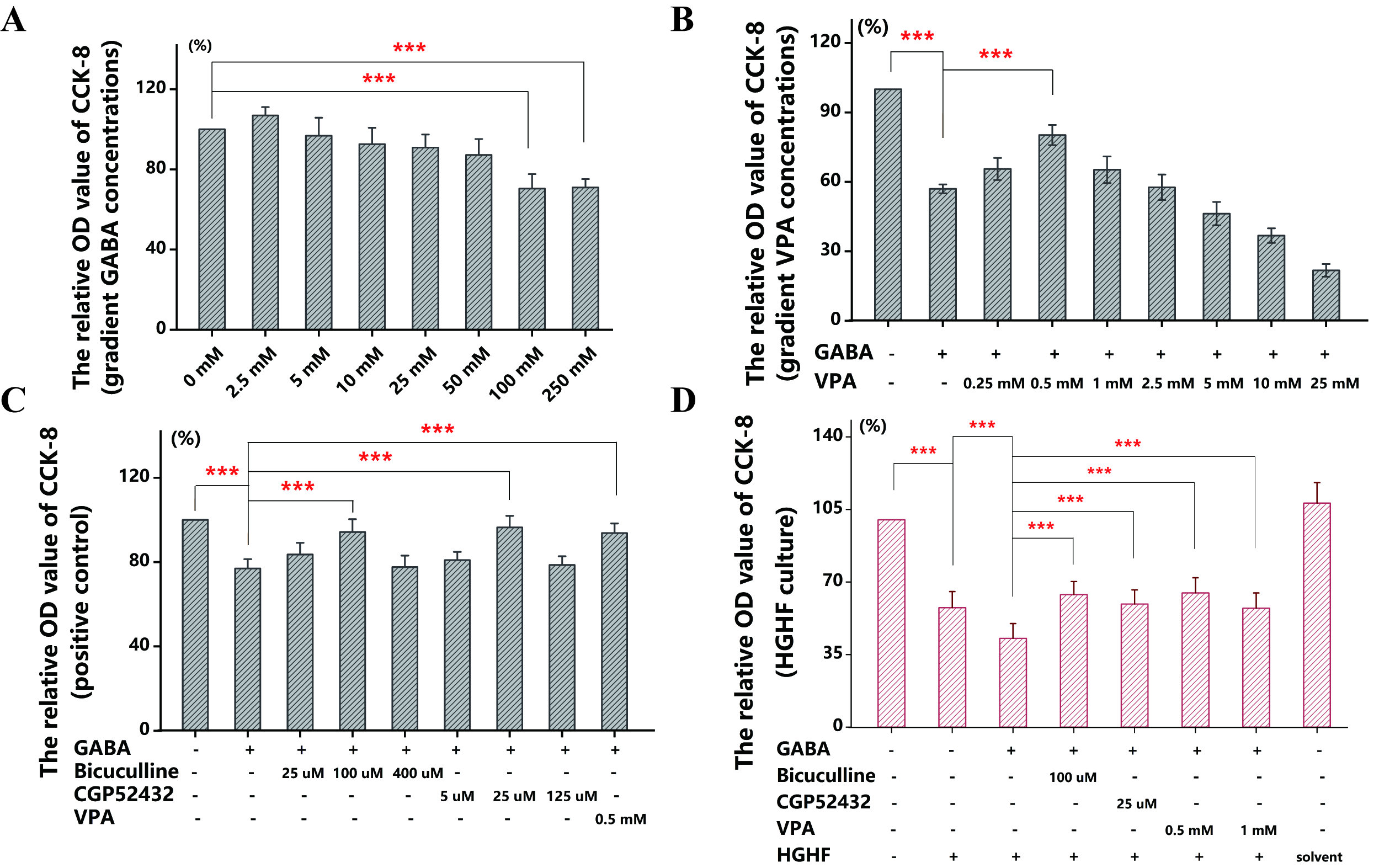

Supplement: Supplementary file 2 — Supplementary Figure 2 [file 41398_2025_3450_MOESM2_ESM.tif]

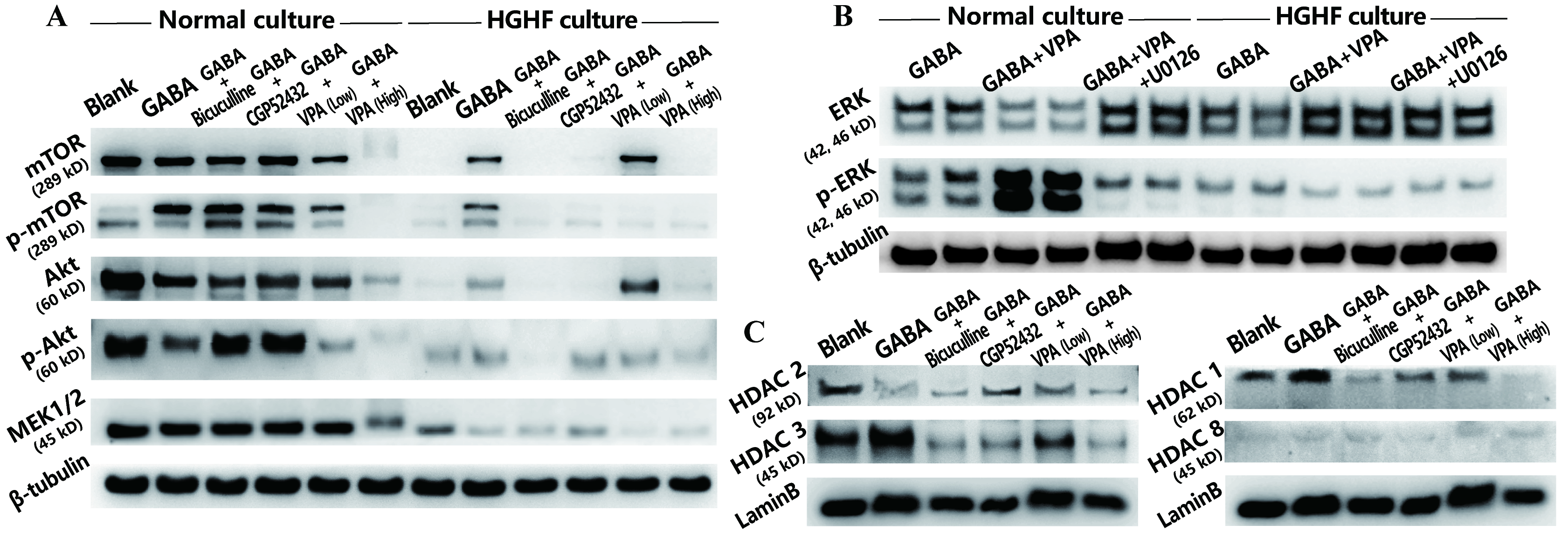

Supplement: Supplementary file 3 — Supplementary Figure 3 [file 41398_2025_3450_MOESM3_ESM.tif]
