# Supplementary material for: Prenatal valproic acid on the basis of gestational diabetes also induces autistic behavior and disrupts myelination and oligodendroglial maturation slightly in offspring
Source: Transl Psychiatry. 2025 Aug 7;15:271. doi: 10.1038/s41398-025-03450-z (PMC12332004; doi:10.1038/s41398-025-03450-z)
Supplement: Supplementary file 4 — Supplementary Figure Legends and Research Elements [file 41398_2025_3450_MOESM4_ESM.docx]

# Supplementary material

## Supplementary Fig.1

**(A)** The fasting blood glucose from PND 28 to PND 56 in four progeny groups: control, VPA, GDM, and GDM+VPA (*n=10*). **(B)** The body weight from PND 14 to PND 56 in the pups of control, VPA, GDM, and GDM+VPA groups (*n=10*). **(C)** The brain morphology after perfusion and fixation in these groups. **(D)** The brain weight of PND 28 mice in four progeny groups (*n=10*). **(E)** The eye-opening scores between PND 8 and PND 12 indicating postnatal brain development in four progeny groups (*n=10*).

## Supplementary Fig.2

**(A)** The OLN-93 was treated with 0 mM, 2.5 mM, 5 mM, 10 mM, 25 mM, 50 mM, 100 mM, or 250 mM GABA in the normal culture for 24 h (*n=6*). The OD value of CCK-8 test was counted to adjust the working concentration of GABA. **(B)** After an 100 mM GABA treatment, the OLN-93 was treated with 0 mM, 0.25 mM, 0.5 mM, 1 mM, 2.5 mM, 5 mM, 5 mM 10 mM, or 25 mM VPA in the normal culture for 24 h (*n=6*). The OD value of CCK-8 test was counted to adjust the working concentration of VPA. **(C)** After an 100 mM GABA treatment, the OLN-93 was treated with 0 μM, 25 μM, 100 μM, or 400 μM (+)-Bicuculline and with 0 μM, 5 μM, 25 μM, or 125 μM CGP52432 in the normal culture for 24 h (*n=6*). The OD value of CCK-8 test was counted to adjust the working concentration of (+)-Bicuculline and CGP52432. **(D)** The OLN-93 was initially treated with 2.5 μM, 5 μM, 10 μM, 20 μM, or 40 μM U0126 for 30 min and with 10 μM U0126 for 15 min or 60 min in the normal culture (*n=6*). After an 100 mM GABA treatment and a 0.5 mM VPA treatment, the OD value of CCK-8 test was counted to adjust the working concentration of U0126.

## Supplementary Fig.3

**(A)** Expression of mTOR, phospho-mTOR, Akt, phospho-Akt and MEK1/2 in the OLN-93 treated with blank, GABA, GABA+Bicuculline, GABA+CGP52432, GABA+VPA (low dose), and GABA+VPA (high dose) in the normal culture and the HFHG culture was evaluated by Western blotting (*n=4*). β-tubulin expression was served as an internal control. **(B)** Expression of ERK and p-ERK in the OLN-93 treated with blank, GABA, GABA+VPA, and GABA+VPA+U0126 was evaluated by Western blotting (*n=4*). β-tubulin expression was served as an internal control. **(C)** Expression of HDAC1, 2, 3, and 8 in the OLN-93 treated with blank, GABA, GABA+Bicuculline, GABA+CGP52432, GABA+VPA (low dose), and GABA+VPA (high dose) in the normal culture was evaluated by Western blotting (*n=4*). LaminB expression was served as an internal control.

# Research Elements

## The preparation of high-fat and high-glucose culture

Palmitic acid (Selleck, S3794) was completely dissolved into100 mM NaOH and turned into a 40 mM palmitic acid solution after full saponification of at 70 °C for 30 min. It was then mixed with 40 mM fat-free BSA (Biofroxx, S3794) at a ratio of 1:1 to obtain the 20 mM palmitic acid solution that can be utilized by OLN-93. The high-fat and high-glucose culture consisted of 1% 20 mM palmitic acid solution, 1% 5 M glucose solution, 1% penicillin-streptomycin premix, 10% fetal bovine serum and 87% DMEM.

## Mice behavioral testing steps

The eye-opening score and the orientation test were used to evaluate the development of pups. Continuous eye opening was observed and scored (closed eyes: 0 points, partial open eyes: 1 point, completely open eyes: 2 points) from PND 8 to PND 12 in each group. The orientation tendency test was performed at PND 12. A 30° slope was established and the pups were placed at the centre of the slope with the head facing sideways down. The time for pups to turn and face the top of the slope was recorded as the orientation tendency test score.

The pups at PND 28 were put in a 50×50×30 cm open field experimental box to acclimatize for 10 min. The cumulative frequency and time of grooming, sniffing, and climbing the sidewall were recorded in the next 10 min. Later, the pups were taken out and kept in isolation for 1 h. Two paired pups from the different nest belonging to the same group were placed in the open field at the same time. During the next 10 min, the cumulative frequency and time of sniffing each other (nose-to-nose, nose-to-body) and moving towards each other (following, pushing, and crawling together) were recorded. In the three-chamber sociability test, PND 56 pups were placed in the middle room of the 40×60×23 cm three-chamber test box for 5 min. Upon opening the channels on both sides, the mice were watched for 30 min in three stages (10 min/stage). In the first stage, the mice were observed for their motor ability and position preference. In the second stage, an stranger mouse and an object were put in the rooms on either side to observe social preference. In the third stage, a new stranger mouse from a different nest replaced the object to record social preference again.

## The mice brain coordinates

Referring to the Allen mouse brain common coordinate framework, we took the anterior forceps at Bregma 1.98 to 1.70 mm, the genu at Bregma 1.10 to 0.86 mm, the body at Bregma 0.74 to 0.02 mm, the splenium at Bregma -1.94 to -2.50 mm, and the posterior forceps at Bregma -2.70 to -2.92 mm in coronal slices of the corpus callosum.

## LFB staining steps

The sections were placed in the pre-heated 0.1% luxol fast blue staining solution (LFB, Solarbio, G3245) after being immersed in ddH_2_O for 3 min. Two hours later, when the sections cooled naturally, they were removed from the staining solution and flushed with ddH_2_O for 3 min. The cortex grew grayish-white after repeating 1 min of 0.05% lithium carbonate solution (Solarbio, G3245) and 10 seconds of 70% ethanol. The sinsed sections were cleaned with the xylene for 10 seconds and were covered with the permount^TM^ mounting medium (Servicebio, G1404).

## Sample preparation steps for TEM

After flushing with a 10 mM PBS, the block from the genus of corpus callosum were fixed in a 1% osmiic acid solution at 4 °C and protected from light for 2 h. After rinsing with 10 mM PBS overnight, They were fixed in an acetone gradient at 4 °C. In a mixture of the epoxy encapsulation solution and acetone, the blocks were immersed overnight. The dry blocks were embedded in pure epoxy resin embedding solution for 2 h, kept at 36 °C for 12 h, kept at 48 °C for 12 h, and polymerized at 60 °C for 48 h. After trimming the blocks, 1 pm slices were cut by a conventional semi-lightning slicer, and localized by toluidine blue staining. The ultrathin slices were cut into 70 nm slices and then spliced onto a copper mesh. After staining with 2% hydrogen peroxide acetate solution for a 15 min, the clean-washed slices were stained with lead citrate solution for 10 min.

## Immunohistochemical staining steps

The sections were dewaxed and rehydrated as the methods described in LFB staining. After being heated in the improved sodium citrate antigen retrieval solution at 95 °C for 20 min and naturally cooling, the hydrogen peroxide antigen closure (15 min) and the secondary antibody incubation of the sections (25 min) were done by the universal mouse/rabbit polymer method detection system (ZSGB-bio, PV-6000). The sections were incubated in the BSA-diluted primary antibodies for 16 h at 4 °C. The chromogenic reaction for 5 min was performed by the DAB chromogenic kit (ZSGB-bio, ZLI-9019, China). After washing in running water, the sections were stained in hematoxylin solution (Beyotime, C0107) for 1 min and then rinsed under running water for 5 min. The sections were treated with the acid alcohol fast differentiation solution (Beyotime, C0163M) for 3 seconds and then rinsed under running water for 5 min again. They were dehydrated by gradient alcohol (75%, 95%, 100%), cleaned with the xylene, and covered with the permount^TM^ mounting medium.

## Immunofluorescent staining steps

After three washes with 10 mM PBS, they were immersed in the quick antigen retrieval solution for frozen sections (Beyotime, P0090) for 10 min. Non-specific antigens were blocked by immersing in 10% goat serum solution (Solarbio, SL038) for 1 h after three washes with 10 mM PBS. The incubation process involved 3% BSA diluted primary antibodies. After incubating at 4 ℃ for 24 h, the slices were rinsed five times with the 10 mM PBS. The mixed secondary antibodies were used to incubate the sections for 1 h at room temperature in the dark. After rinsing with the 10 mM PBS, the sections were affixed to slides and covered with antifade mounting medium with DAPI (Beyotime, P0131).

## Immunoblotting steps

The samples and the marker (Thermo Fisher Scientific, 26616) were added to the wells of BeyoGel^TM^ plus precast PAGE gel (Tris-Gly, 8%, 15 wells, Beyotime, P0452). The proteins were electrophoresed for 75 minutes at 100 V in the BeyoGel^TM^ plus SDS-PAGE hepes electrophoresis buffer (Beyotime, P0552). They were transferred to 0.2 μm PVDF membranes (Millipore, ISEQ00010) at 0.25 mA for 90 min in the western rapid transfer buffer (Beyotime, P0572). The bands were immersed in the Wb antigen blocking and antibody sensitization diluent (Willget-bio, F01) for 30 min and were incubated in primary antibodies diluted with 5% BSA for 24 hours. They were rinsed with TBS contained 0.1% Tween-20 were incubated with HRP-labeled goat anti-rabbit IgG (1:3000, Beyotime, A0208) or HRP-labeled goat anti-mouse IgG (1:3000, Beyotime, A0216) for a hour. Using ImageQuant (GE, LAS4000, America), the images were captured with the assistance of BeyoECL star kit (Beyotime, P0018AS).
